# Cannabinoids for the prevention of chemotherapy-induced nausea and vomiting in oncological therapy: a systematic review

**DOI:** 10.1007/s11764-025-01876-4

**Published:** 2025-08-12

**Authors:** Sarah F. Kemmner, Jennifer Dörfler, Jutta Huebner

**Affiliations:** Klinik Für Innere Medizin II, Hämatologie Und Internistische Onkologie, Universitätsklinikum Jena, Am Klinikum 1, Jena, 07747 Germany

**Keywords:** Cannabinoids, Cancer treatment, Nausea, Vomiting

## Abstract

**Purpose:**

Cannabinoids are compounds that occur naturally in cannabis plants. The objective of this systematic review was to provide an overview of the existing evidence regarding the use of cannabinoids for the management of chemotherapy-induced nausea and vomiting (CINV) in cancer patients.

**Methods:**

A systematic literature search was conducted in June 2024 in five electronic databases (Embase, Cochrane, PsychInfo, CINAHL, and Medline) to identify studies examining the utilization, efficacy, and potential adverse effects of cannabinoid-based therapy in cancer patients.

**Results:**

A total of 32 studies comprising 1889 patients suffering from different cancer types were included in the systematic review. Cannabinoid-based therapy involved the oral administration of defined and approved cannabis preparations. Of the 22 studies comparing cannabinoid-based CINV management to now outdated antiemetic therapies, 12 found a significant benefit of cannabinoids in CINV management, and eight out of nine studies comparing cannabinoids to placebo observed such a benefit. Only one of the 32 studies with a risk of bias compared cannabinoids to guideline-based therapy with a 5-HT3 receptor antagonist, an NK1 receptor antagonist, and dexamethasone and demonstrated a benefit of cannabinoids in reducing nausea and vomiting, but the therapy was associated with cannabinoid-related side effects.

**Conclusion:**

With only one study complying with modern antiemetic standards and considering the risks of side effects, clinical evidence to recommend cannabinoids for the management of CINV is deemed insufficient.

**Implications for Cancer Survivors:**

Cancer patients suffering from CINV might find alleviation using cannabinoids, but heterogenous results and side effects prevent clinical recommendation.

**Supplementary Information:**

The online version contains supplementary material available at https://doi.org/10.1007/s11764-025-01876-4.

## Introduction

Cannabinoids are compounds primarily found in cannabis plants (*Cannabis sativa* and *Cannabis indica*). Over 144 cannabinoids, including delta-9-tetrahydrocannabinol (THC) and cannabidiol (CBD), have been identified in cannabis. THC is a psychoactive component, whereas CBD, another major cannabinoid, is nonpsychoactive and has been suggested to exhibit analgesic, anti-inflammatory, calming, and anxiolytic effects. Synthetic cannabinoids, such as nabilone, are man-made derivatives of THC that also possess psychoactive properties. Cannabis-based medications have been used in the treatment of neuropathic pain and spasticity (nabiximols), appetite loss in patients with HIV/AIDS, and chemotherapy-induced nausea and vomiting (nabilone, dronabinol). Many cancer patients suffer from chemotherapy-induced nausea and vomiting (CINV), which can be classified into three distinct categories. These include anticipatory (occurring prior to chemotherapy administration), acute (present within 24 h following chemotherapy), and delayed (more than 24 h after chemotherapy) CINV [1].

Nausea and vomiting constitute prevalent side adverse effects of chemotherapy. These symptoms significantly impact patients’ quality of life, compromise treatment adherence, and can lead to additional health complications.

Antiemetic treatment improved after the development of serotonin (5-HT_3_) antagonists in the early 1990 s, and the use was potentiated by a concomitant use of corticosteroids to control emesis [2, 3]. Despite this progress, antiemetic control often remains suboptimal [2, 4, 5]. For decades, cannabinoids have been discussed as a potential additional treatment in cases of CINV.

Therefore, this systematic review was conducted with the objective of assess and evaluating potential positive and negative effects of cannabinoids on the clinical course of cancer chemotherapy.

## Methods

### Inclusion and exclusion criteria

The inclusion and exclusion criteria are listed in Table 1 and are based on the PICO (P, patient/problem; I, intervention; C, comparison intervention; O, clinical outcome) model [6]. Randomized controlled trials (RCTs) were included, if they reported patient-relevant outcomes after the treatment of adult cancer patients with any interventions containing cannabinoids. Because of the wide range of application fields, all cancer entities and all types of chemotherapy (low and high emetic potential) were included. The criteria for rejecting studies encompassed primary prevention, gray literature, publication types other than primary investigations/reports (e.g., comments, letters, abstracts), and study populations with precancerous conditions. Additionally, studies were excluded if they reported no patient-centered outcomes (such as laboratory parameters). Languages were restricted to English and German.

**Table 1:** Inclusion and exclusion criteria

PICO	Inclusion criteria	Exclusion criteria
Patient	Cancer patients (all entities and stages) At least 80% of patient population constitutes adult patients (age ≥ 18)	Patients with precancerous conditions or carcinoma in situ Preclinical studies Study population with more than 20% of pediatric patients or patients with precancerous conditions
Intervention	Any intervention with cannabinoids	
Comparison	All types of control groups (placebo, standard care, observation)	Other study types (one-armed/non-controlled studies, case report or series)
Outcome	Symptoms of nausea and vomiting independent of cause and toxicity	No patient- centered data, for example laboratory parameters (except PSA)
Other criteria	RCTs Languages: German and English as full publications	Gray literature (conference articles, abstracts, letters, ongoing studies, unpublished literature…)

### Study selection

In June 2024, a systematic research was conducted with five databases (Medline (Ovid), CINAHL (EBSCO), EMBASE (Ovid), Cochrane CENTRAL, and PsycINFO (EBSCO)). For each of these databases, a complex search strategy was developed consisting of a combination of MeSH terms, keywords, and text words in different spellings connected to cancer and cannabinoid-based therapy (Table S1). After the search results were imported into EndNote 20, all duplicates were removed, and a title-abstract screening was carried out by two independent reviewers (SK, JD). In cases of disagreement, a third reviewer was consulted (JH), and consensus was reached by discussion. All the full texts were subsequently retrieved and screened independently by both reviewers. When the title and abstract did not have sufficient information for screening purposes, a full-text copy was obtained. Additionally, the bibliography lists of all the retrieved articles were searched for relevant cannabinoid studies.

### Assessment of risk of bias and methodological quality

All characteristics were assessed by two independent reviewers (SK, JD). In cases of disagreement, a third reviewer was consulted (JH), and consensus was reached by discussion.

The risk of bias in the included studies was analyzed with the Cochrane revised Risk of Bias Tool 2.0 [7]. Additional criteria concerning methodology were population size, power analysis application, and the statistical adequacy of the test.

### Data extraction

Data extraction was performed by one reviewer (SK) and supervised by two independent reviewers (JD, JH). As a template for data extraction, evidence tables from the National Guideline on Complementary and Alternative Medicine in Oncological Patients of the German Guideline Program in Oncology [8, 9] were used. SRs were examined for primary literature that met the inclusion criteria of this study.

## Results

**Fig. 1:**
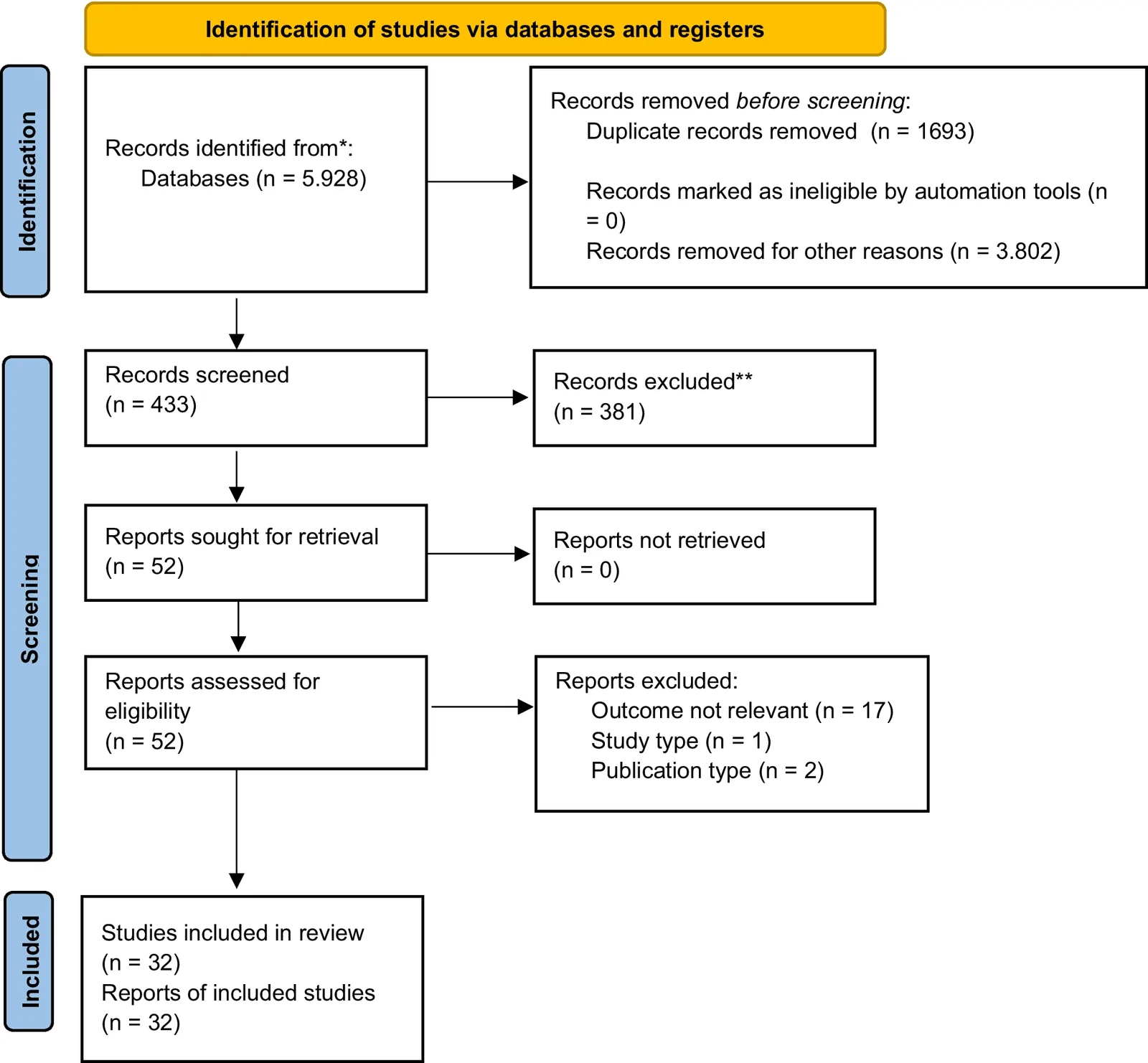
Consort diagram. *Consider, if feasible, reporting the number of records identified from each database or register searched (rather than the total number across all databases/registers). **If automation tools were used, indicate how many records were excluded by a human and how many were excluded by automation tools. *From:* Page MJ, McKenzie JE, Bossuyt PM, Boutron I, Hoffmann TC, Mulrow CD, et al. The PRISMA 2020 statement: an updated guideline for reporting systematic reviews. *BMJ* 2021;372:n71. https://doi.org/10.1136/bmj.n71. For more information, visit: http://www.prisma-statement.org/

The systematic research revealed 5928 results. In a first step, duplicates (*n* = 1693) were removed, and 3802 studies were removed for other reasons, leaving 433 studies for screening After screening the titles and abstracts, 52 studies remained to be reviewed in depth (Fig. 1). Finally, 32 publications were included to create this review.

### Characteristics of included studies

Among the selected studies, a total of 1889 patients were included and 1495 patients were analyzed. The age of the patients ranged from nine to 82 years. All studies included at least 80% of participants over the age of 18, with the exception of studies that lacked this information. As these studies were not specifically designated as pediatric studies, it was assumed these participants were also over the age of 18. A total of 711 participants were female, 780 were male, and 398 had no sex listed. Guideline-compliant prophylaxis for high emetogenic chemotherapy includes the use of 5-HT3 receptor antagonists, NK1 receptor antagonists, and dexamethasone. Among the included studies, only one met these criteria [10]. We included 31 RCTs that followed traditional antiemetic standards [11–41], encompassing three studies that used new antiemetics (5-HT3 receptor antagonist) but did not adhere to guideline-recommended combinations [39–41] (Table 2).

**Table 2:** Characteristics included studies

Reference	Study type	*n*/cancer type/dropout	Intervention/duration	Endpoints	Outcomes
Sukpiriyagul et al. (2023)	RCT, crossover	***n*** = 60; **Drop out:** *n* = 6 **Cancer type:** Ovarian cancer, endometrial cancer, cervical cancer, others	**Intervention:** THC:CBD, oil, 1 ml contains: 2.7 mg THC and 2.5 mg CBD **Comparison:** Placebo **Route of administration:** orally **Regime:** Days 1–3: 1 drop in the morning and 1 drop in the evening before meals; days 4–5: 1 drop in the morning, at noon, and in the evening before meals	T0: Baseline T1: Cycle 1 (5 days) T2: Cycle 2 (5 days) Crossover: implemented after 5 days (after T1) 1. Nausea (by self-administered questionnaire, scale 0–10: 0 = no nausea, 10 = worst possible nausea)	1. Mean: day 1: THC:CBD 2.019, placebo 3.113, *p* = 0.001, sign.; day 2: THC:CBD 2.189, placebo 3.038, *p* = 0.007, sign.; day 3: THC:CBD 2.434, placebo 2.962, *p* = 0.097, n.s.; day 4: THC:CBD 2.094; placebo 2.717, *p* = 0.029, sign.; day 5: THC:CBD 1.830; placebo 3.151, *p* < 0.001, sign; total: THC:CBD 2.113 (95% CL: 1.940, 2. 315), placebo 2.996 (95% CL: 2.795, 3.197), *p* = 0.001, sign
Grimison et al. (2020)	RCT, crossover	***n*** = 81 **Drop out**: *n* = 9 **Cancer type:** Breast, colorectal, lung, esophageal/gastric, gynecological, pancreatic, hematological, testicular, others	**Intervention:** THC 2.5 mg, CBD 2.5 mg **Comparison:** Placebo **Route of administration:** orally **Regime:** Three times daily, 1 day before chemotherapy until day 5	T0: Baseline T1: 1 day before chemotherapy T3: day 8 of each cycle T4: between 30 and 42 days after the end of the intervention Crossover: after one cycle of chemotherapy, results were assessed after two cycles 1. CINV CR (no significant nausea, no vomiting or rescue medication from 0 to 120 h after chemotherapy) 2. Self-reported CR (“no vomiting, no clinically significant nausea,” defined as nausea < 2 on a 10-point scale, and “no use of rescue medications”)	1. Sign. advantage for THC/CBD (25%) compared to placebo (14%), RR 1.77, 90% CI 1.12–2.79, *p* = 0.041 2. CR: THC/CBD: *n* = 18 (25%), placebo *n* = 10 (14%), absolute difference: 11%, RR 1.8, Cl 1.1–2.8, sign. advantage for THC/CBD, *p* = 0.04; no vomiting: THC/CBD: *n* = 50 (69%), placebo *n* = 41 (57%), absolute difference: 12.5%, RR 1.2, Cl 1.0–1.4, *p* = 0.05, ns.; no use of rescue medications: THC/CBD: *n* = 20 (28%), placebo *n* = 11 (15%), absolute difference: 12.5%, RR 1.8, Cl 1.1–2.8, *p* = 0.04, sign.; no sign. nausea score (score < 2): THC/CBD: *n* = 15 (21%), placebo *n* = 7 (10%), absolute difference: 11%, RR 2.0, Cl 1.2–3.4, *p* = 0.03, sign.; CR and no sign. nausea**:** THC/CBD: *n* = 9 (13%), placebo *n* = 4 (6%), absolute difference: 7%, RR 2.1, Cl 0.96–4.8, *p* = 0.12, ns.; mean number of vomits per day ± SD: THC/CBD: *n* = 0.2 ± 0.0, placebo *n* = 0.6 ± 0.2, absolute difference: − 0.4, Cl − 0.7 to − 0.2, *p* = 0.003, sign.; maximum number of vomiting per day (mean ± SD): THC/CBD: *n* = 0.5 ± 0.1, placebo *n* = 1.4 ± 0.3, absolute difference: − 0.8, Cl − 1.2 to − 0.4, *p* = 0.001, sign.; mean nausea score (mean ± SD): THC/CBD: *n* = 3.2 ± 0.2, placebo *n* = 4.7 ± 0.2, absolute difference: − 1.4, Cl − 1.8 to − 1.0, *p* < 0.001, sign.; maximum nausea score (mean ± SD): THC/CBD: 4.3 ± 0.3, placebo 6.1 ± 0.3, absolute difference: − 1.8, Cl − 2.3 to − 1.2, *p* < 0.001, sign
Duran et al. (2010):	RCT	***n*** = 16 **Drop out**: *n* = 1 **Cancer type:** Breast, ovary, lung	**Intervention:** THC and CBD, each spray delivered 2.7 mg THC and 2.5 mg of CBD **Comparison:** Placebo **Route of administration:** Oromucosal spray **Regime:** Day 0: Following administration of chemotherapy (at time points 0, 30, and 120 min); days 1–4: gradual dose escalation to a maximum of ≤ 8 sprays within a 4-h interval, mean daily dose: 4.8 sprays	T0: Baseline T1: Day 0 T2: Day 1 T3: Day 2 T4: Day 3 T5: Day 4 1. CR: defined as no vomiting and average nausea VAS score < 10 mm 2. PR: defined as vomiting 1–4 times daily and average nausea VAS score < 25 mm 3. Delayed emesis and nausea 4. Severity and duration of nausea and vomiting	1. overall period (0–120 h): THC/CBD *n* = 5/7, 71.4% compared to placebo *n* = 2/9, 22.2%, representing a difference of 49.2%, no *p* values provided, sign.; acute period (0–24 h): THC/CBD *n* = 5/7, 71.4% vs. placebo *n* = 6/9, 66.7%, no *p* value provided, n.s.; delayed period (24–120 h): THC/CBD *n* = 5/7 71.4% vs. placebo *n* = 2/9, 22.2%, no *p* value reported, sign 2. THC/CBD *n* = 1, placebo *n* = 5, no *p* value reported 3. No delayed emesis T2 and T5: THC/CBD *n* = 7 (71.4%), placebo *n* = 2 (22.2%), difference (49.2%), 95% CL: 1.0, 75.0, THC/CBD demonstrated a significantly superior outcome, no *p* value reported; no delayed nausea: THC/CBD *n* = 4 (57.1%), placebo *n* = 2 (22.2%), difference (34.9%), 95% CL: − 10.8, 66.3, no *p* value reported; no significantly delayed nausea: THC/CBD *n* = 5 (71.4%), placebo *n* = 4 (44.4%), difference (27.0%), 95% CL: − 18.0, 59.7, no *p* value reported 4. THC/CBD showed trends toward improved severity and duration of nausea and vomiting, n.s., no *p* value reported
Meiri et al. (2007)	RCT	***n*** = 64 **Drop out:** *n* = 12 **Cancer type:** Breast, non-small cell lung cancer, lung, ovarian, prosate, other small cell cell cancers, liver, kidney, pancreatic, Hodgkins, nonHodgkin’s, bladder	**Intervention:** Dronabinol, 2.5 mg **Comparison:** Ondansetron, 8 mg **Comparison:** Dronabinol + ondansetron **Comparison:** Placebo **Route of administration:** Orally **Regime:** On day 1, all groups received standard antiemetic therapy; on day 2 D: QID, O: BID, O + D: D QID and O BID; on days 3–5, a flexible dosing regimen was initiated	T0: Baseline Tag 1 (CAT vs. placebo) T1: Tag 2 T2: Tag 3 T3: Tag 4 T4: Tag 5 T5: Tag 6,7 oder 8 follow-up 1. Complete response (nausea and vomiting) with VAS 2. Nausea (absence of nausea) 3. Mean nausea intensity with VAS 4. Mean episodes of vomiting/retching 5. Duration of nausea	1. T0: Combined active treatment (79%) vs. placebo (40%), *p* = 0.024 sign.; T1–T4: dronabinol (54%), ondansetron (58%), combination (47%), placebo (20%), active treatments vs. placebo n.s.; exploratory analysis reveals a statistically significant better response with ondansetron compared to placebo (58% vs. 20%, *p* = 0.04) 2. T0: Combined active treatment 79%, placebo 38%, *p* = 0.013; T1–T4: dronabinol 71%, ondansetron 64%, combination 53%, placebo 15%, active treatment groups vs. placebo *p*’ < 0.05, sign; differences between active treatment groups, n.s 3. T0: Combined active treatment 7.65%, placebo 30.67%, *p* = 0.029; T1–4: dronabinol 10.1%, ondansetron 24.0%, combination 14.3%, placebo 48.4%, active treatment groups vs. placebo, *p*’ < 0.05, n.s. differences between active treatment groups 4. T1: Dronabinol 0.1, ondansetron 0.7, combination 0.4, placebo 0.4; T2: dronabinol 0.3, ondansetron 0.3, combination 0.9, placebo 1.6; T3: dronabinol 0.1, ondansetron 0, combination 0, placebo 0; T4: dronabinol 0, ondansetron 0.7, combination 0, placebo 0; days 2–5 (LOCF): dronabinol 0.2; ondansetron 1.3; combination 0.7; placebo 1.3, no *p* values reported, n.s 5. T1–T4: Comparable across groups, no values reported, no *p* value reported
Lane et al. (1991)	RCT	*n* = 62; Drop out: *n* = 23 Cancer type: Breast, colon, lymphoma, miscellaneous	Intervention: Dronabinol (Marinol—10 mg) and placebo Comparison: Prochlorperazine 10 mg and placebo Comparison: Prochlorperazine 10 mg and dronabinol 10 mg Route of administration: Orally Regime: Treatment regimens up to 5 days, treatment was administered every 6 h	T0: Baseline T1: Day 0: anticipatory T2: Days 1–5: chemotherapy-induced 1. Presence of nausea and vomiting (T1, T2) 2. Duration of nausea and vomiting (median, with VAS) 3. Severity of nausea with VAS	1. T1: Before chemotherapy (anticipatory): *n* = 11, 30% of patients treated with dronabinol, 0% treated with prochlorperazine, and 26% in the combination group experienced at least one episode of nausea, dronabinol vs. prochlorperazine: *p* < 0.05, sign., prochlorperazine vs. combination *p* < 0.05, sign., no anticipatory vomiting with prochlorperazine and combination treatment, 1 patient with dronabinol; 2. T2: During chemotherapy: no nausea and vomiting with dronabinol (41%), prochlorperazine (30%), combination (47%), n.s., no *p* value reported 3. T2: Significantly shorter with the combination (2 min) vs. dronabinol (5 min) and prochlorperazine (5 min), *p*’s <.001; duration of nausea alone: dronabinol (10 min) vs. prochlorperazine (15 min), n.s. no *p* value not readable; duration of nausea alone: dronabinol (2 min) vs. prochlorperazine (4 min), n.s., *p* value not readable 4. T2: Significantly less severe nausea with the combination compared to dronabinol and prochlorperazine, *p*’s < 0.001; other *p*’s not readable
McCabe et al. (1988)	RCT, crossover	***n*** = 36; **Drop out: *n* = **0 **Cancer type:** Breast, hematologic malignancies, melanoma, ovarian, testicular, gastrointestinal, sarcoma	**Intervention:** THC 15 mg/m2 **Comparison:** Prochlorperazine 10 mg **Route of administration:** Orally **Regime:** 1 h before chemotherapy and every 4 h after chemotherapy for a total of 24 h	T0: Baseline T1: Chemotherapy (24 h) T2: Chemotherapy (24 h) T3: Chemotherapy (24 h) T4: Chemotherapy (24 h) Each patient was randomly assigned to receive each intervention twice in a crossover sequence of four chemotherapy cycles 1. CR = complete absence of nausea and vomiting. (Assessment criteria: nausea rated on a scale of 0 = none to 4 = unacceptable, CR = complete absence of nausea and vomiting) 2. PR = at least 50% reduction in nausea and vomiting compared to T0	1. THC *n* = 9 (25%), prochlorperazine *n* = 0, no *p* value reported 2. THC *n* = 14 (39%), prochlorperazine *n* = 1, no *p* value reported
Pomeroy et al. (1986)	RCT	***n*** = 38; **Drop out: *n* = **7 **Cancer type:** Ovary, testis, bronchus, nonHodgkin’s lymphoma, Hodgkin’s disease, sarcoma, breast, melanoma, nephroblastom	**Intervention:** Nabilone 1 mg **Comparison:** Domperidone 20 mg **Route of administration:** Orally **Regime:** In the night before chemotherapy and every 8 h during the day of chemotherapy	T0: Baseline T1: Chemotherapy cycle 1 T2: Chemotherapy cycle 2 1. Mean number of vomiting episodes 2. Nausea (mean, scores 1–3)	1. T1 mean: Nabilone 4.76, domperidone 12.95, *p* < 0.02, sign; T2 mean: nabilone 4.27, domperidone 7.69; *p* > 0.10, n.s.; T1 and T2 mean: nabilone 4.53, domperidone 10.81, *p* < 0.01, sign 2. T1 mean: Nabilone 1.47, domperidone 2.05, *p* > 0.05, n.s.; T2: mean: nabilone 1.53, domperidone 1.93, *p* > 0.05, n.s., T1 and T2 mean number: nabilone1.5; domperidone 2.0, *p* > 0.05, n.s
Niederle et al. (1986)	RCT, crossover	***n*** = 20; **Drop out: *n* = **10 **Cancer type:** Testicular	**Intervention:** Nabilone 2 mg **Comparison:** Alizaprid 150 mg **Route of administration:** Orally **Regime:** In the night before chemotherapy, and on days 1–5: 8 a.m. and 6 p.m	T1: Cycle 1 (5 days) T2: Cycle 2 (5 days) Crossover: The crossover occurred following the completion of one cycle of chemotherapy 1. Nausea (questionnaire, none/mild = no impact on normal activity, moderate = impact on normal activity, severe = bedridden > 12 h) 1. Vomiting (episodes)	1. Frequency and severity: Lower in each period with nabilone vs. alizaprid, no mean values reported, *p* < 0.01, sign.; average duration: nabilone 1.3 vs. alizaprid 5.1 h, *p* < 0.01, sign 2. Daily mean episodes: Nabilone 1.1 vs. alizaprid 2.9, *p* < 0.01, sign
Crawford et al. (1985)	RCT, crossover	***n*** = 32; **Drop out: *n* = **1 **Cancer type:** Adenocarcinoma of the ovary, germ cell tumor	**Intervention:** Nabilone 1 mg (capsule) + placebo (intravenously) **Comparison:** Metoclopramide 1 mg kg^−1^ in 100 ml 0.9% NaCI over 15 min (intravenously) **Route of administration:** Orally + intravenously **Regime:** Nabilone/placebo: on rising 1 capsule, after 2 h 2 capsules and on retiring, every 8 h as needed; metoclopramide: 30 min before chemotherapy, 90 min after, 3.5 h after, 6.5 h after and every 3 h as required	T0: Baseline T1: After 2 cycles of chemotherapy T2: After 4 cycles of chemotherapy Crossover: A crossover was conducted following the completion of one chemotherapy cycle, with three instances of crossover 1. Nausea (questionnaire, scale (1–10): no nausea = 0, nausea all the time = 10) 2. Vomiting (episodes, volume)	1. T2, within-patient comparison: nabilone vs. metoclopramide, no mean values reported, *p* > 0.05, n.s.; between-patients comparison mean: nabilone 5.76, metoclopramide 5.63, *p* > 0.05, n.s 2. T2, within-patient comparison: number of vomiting episodes: nabilone vs. metoclopramide, *p* > 0.5, n.s.; mean volume of vomit: nabilone vs. metoclopramide, *p* > 0.1, n.s.; number of patients who had < 3 episodes in one round but ≥ 3 in the other: nabilone vs. metoclopramide, *p* > 0.3, n.s.; between-patients comparison: mean number of vomiting episodes: nabilone 7.86 vs. metoclopramide 6.13, *p* > 0.1, n.s.; mean volume of vomit: nabilone vs. metoclopramide, nabilone 681.51 ml, metoclopramide 536.66 ml, *p* > 0.1, n.s.; % of cycles with < 3 episodes: nabilone 10.8, metoclopramide 30.8, *p* < 0.05, sign
Niiranen et al. (1985)	RCT, crossover	***n*** = 32; **Drop out: *n* = **8 **Cancer type: lung cancer**	**Intervention:** Nabilone 1 mg **Comparison:** Prochlorperazine 7.5 mg capsules **Route of administration:** Orally **Regime:** Two chemotherapy cycles were compared, 1 cycle comprises 28 days, administration: Two times before chemotherapy, 12-h intervals up to 24 h after chemotherapy	T0: Baseline T1: Cycle 1 (28 days) T2: Cycle 2 (28 days) Crossover: after one cycle of chemotherapy (28 days) 1. Severity of nausea with a homemade questionnaire 2. Vomiting episodes 3. Efficacy with a homemade questionnaire	1. T2 and T1: none: nabilone *n* = 1, prochlorperazine *n* = 4; mild: nabilone *n* = 7, prochlorperazine *n* = 4; moderate: nabilone *n* = 9, prochlorperazine *n* = 10; severe: nabilone *n* = 7, prochlorperazine *n* = 6; n.s., no *p* value, 2. T2 and T1mean: nabilone 6.5 compared to prochlorperazine: 11, *p* < 0.05, sign 3. T2 and T1: very good: nabilone *n* = 3, prochlorperazine *n* = 5; good: nabilone *n* = 9, prochlorperazine *n* = 3; fair: nabilone *n* = 5, prochlorperazine *n* = 6; poor: nabilone *n* = 6, prochlorperazine *n* = 3; very poor: nabilone *n* = 1, prochlorperazine *n* = 7; no *p* value reported, n.s
Sheidler et al. (1984)	RCT, crossover	***n*** = 20; **Drop out:* n* = **4 **Cancer type:** small cell lung cancer, multiple myeloma, ovarian cancer, adenocarcinoma of the lung, breast cancer, diffuse histocytic lymphoma, rhabdomyosarcoma	**Intervention:** levonantradol 1 mg **Comparison:** Prochlorperazine 10 mg **Route of administration:** Intramuscularly **Regime:** One treatment course each, 2 h before chemotherapy and 2, 4, and 6 h after chemotherapy	No timeline given 1. Complete response, partial response, no response with a homemade score (nausea: 1 none-5 bedridden > 2 h, vomiting: 1 none- 5 severe) 2. Subgroup comparison (previously undergone chemotherapy vs. first-time chemotherapy)	1. Complete response: levonantradol *n* = 1, prochlorperazine *n* = 2, with levonantradol and prochlorperazine *n* = 1; partial response: levonantradol *n* = 9, prochlorperazine *n* = 9, with levonantradol and prochlorperazine *n* = 6; no response: levonantradol *n* = 6, prochlorperazine *n* = 5, with levonantradol and prochlorperazine *n* = 2; response to prochlorperazine and levonantradol *p* = 0.6, n.s., 2. Previously undergone *p* = 1.0, first-time: *p* = 0.51; n.s
Heim et al. (1984)	RCT, crossover	***n*** = 57; **Drop out: *n* = **12 **Cancer type:** Lung, lymphoma, soft-tissue sarcoma, breast, testis, melanoma, ovary, osteosarcoma, prostatecancer, head and neck	**Intervention:** Levonantradol, 0.5 mg **Comparison:** Metoclopramide, 10 mg **Route of administration:** Intramuscularly **Regime:** 1 h before and 2 and 6 h after chemotherapy	T0: Baseline T1: After 1 cycle T2: After 2 cycles Crossover: after one chemotherapy cycle 1. Nausea (scale from 1 to 4: 1 none, 2 mild, 3 moderate, 4 severe) 2. Vomiting with a questionnaire 3. Cisplatin subgroup (*n* = 24)	1. T2: 28 patients (62%) had less nausea with levonantradol compared to 5 (11%) with metoclopramide, *p* < 0.05, sign.; equal for 12 patients 2. T2: less vomiting with levonantradol *n* = 25 compared to *n* = 8 with metoclopramide, *p* < 0.05, sign., levonantradol equals metoclopramide in *n* = 12; episodes of vomiting: levonantradol 140, metoclopramide 301, no *p* value 3. Better antiemetic effect with levonantradol, no mean value reported, *p* = 0.0001 (uncorrected), sign
Hutcheon et al. (1983)	RCT, single blind	***n*** = 108; **Drop out: *n* = **36 **Cancer type:** Not given	**Intervention:** Levonandradol 0.5 mg, **Comparison:** Levonandradol 0.75 mg **Comparison:** Levonandradol 1 mg, **Comparison:** Chlorpromazine 25 mg **Route of administration:** Intramuscularly **Regime:** 2 h before chemotherapy, 2 h after chemotherapy, 2 doses in a 4-h interval	T0: Baseline T1: After chemotherapy cycles 1. Nausea (with a scale: none, mild, moderate, severe) 2. Vomiting (episodes)	1. T1: no: 0.5 mg levonantradol* n* = 14, 0.75 mg levonantradol *n* = 8, 1 mg levonantradol *n* = 13, chlorpromazine *n* = 9; mild: 0.5 mg levonantradol *n* = 6, 0.75 mg levonantradol *n* = 14, 1 mg levonantradol *n* = 4, chlorpromazine *n* = 13; moderate: 0.5 mg levonantradol *n* = 07, 0.75 mg levonantradol *n* = 5, 1 mg levonantradol *n* = 6, chlorpromazine *n* = 4; severe: 0.5 mg levonantradol *n* = 0, 0.75 mg levonantradol *n* = 1, 1 mg levonantradol *n* = 3, chlorpromazine *n* = 1, no *p* value reported, n.s 2. T1: 0 episodes: 0.5 mg levonantradol *n* = 20, 0.75 mg levonantradol *n* = 11, 1 mg levonantradol *n* = 14, chlorpromazine *n* = 11; 1–4 episodes: 0.5 mg levonantradol *n* = 3, 0.75 mg levonantradol *n* = 11, 1 mg levonantradol *n* = 4, chlorpromazine *n* = 9**;** 5–10 episodes: 0.5 mg levonantradol *n* = 2, 0.75 mg levonantradol *n* = 5, 1 mg levonantradol *n* = 8, chlorpromazine *n* = 7; 10 episodes: 0.5 mg levonantradol *n* = 2, 0.75 mg levonantradol *n* = 1, 1 mg levonantradol *n* = 0, chlorpromazine *n* = 0; more patients achieved complete relief in groups treated with 0.5 mg levonantradol and 0.75 mg levonantradol compared to chlorpromazine: 0.5 mg levonantradol vs. chlorpromazine *p* < 0.05, sign., 0.75 levonantradol vs. chlorpromazine, *p* < 0.01, sign.,
Ahmedzai et al. (1983)	RCT, crossover	***n*** = 34; **Drop out: *n* = **8 **Cancer type:** lung cancer	**Intervention:** Nabilone 1-mg capsules **Comparison:** Prochlorperazine 5 mg capsules **Route of administration:** Orally **Regime:** Two cycles of chemotherapy, each 21 days, Nabilone or prochlorperazine were administered from day 0 to day 3, day 0: 1 × 2 capsules, days 1–3: 2 × 2 mg nabilone or 3 × 10 mg prochlorperazine	T0: Baseline T1: Cycle 1 (days 1–3 of 21 days) T2: Cycle 2 Crossover: after one chemotherapy cycle (21 days) 1. Symptoms with a selfmade score (0–3) 2. Vomiting episodes	1. Nausea mean**:** day 1: nabilone 0.3, prochlorperazine 1.0, *p* < 0.005, sign.; day 2 mean: nabilone 0.4 prochlorperazine 1.1, *p* < 0.01, sign.; day 3 mean: nabilone 0.1, prochlorperazine 0.6, *p* < 0.05, sign.; retching mean: day 1: nabilone 0.1, prochlorperazine 0.9, *p* = 0.001, sign.; day 2 mean: nabilone 0.2, prochlorperazine 0.9, *p* < 0.01, sign.; day 3 mean: nabilone 0.1, prochlorperazine 0.5, no *p* value, n.s.; vomiting mean: day 1: nabilone 0.3, prochlorperazine 0.7, no *p* value, n.s.; day 2 mean: nabilone 0.3, prochlorperazine 0.9, *p* < 0.05, sign.; day 3 mean: nabilone 0, prochlorperazine 0.6, *p* < 0.001, sign 2. Day 1: nabilone *n* = 6 (22%), prochlorperazine *n* = 9 (30%), no *p* value, n.s.; day 2: nabilone *n* = 4 (15%), prochlorperazine *n* = 13 (43%), *p* = 0.05, n.s.; day 3: nabilone *n* = 0, prochlorperazine *n* = 8 (30%), *p* < 0.01, sign.; episodes per person per day 1: nabilone 1.3, prochlorperazine 3.0, n.s., day 2: nabilone 2.0, prochlorperazine 2.6, *p* < 0.05, sign.; day 3: nabilone 0, prochlorperazine 2.0, *p* < 0.005, sign
Kleinman et al. (1983)	RCT, crossover	***n*** = 16; **Drop out: *n* = **2 **Cancer type:** Not given	**Intervention:** THC 15 mg and prochlorperazine 10 mg **Comparison:** Prochlorperazine 10 mg and placebo **Route of administration:** Orally **Regime:** 1 h before chemotherapy, 4 h after, 8 h after	T0: Baseline T1: Two courses of chemotherapy T2: Two courses of chemotherapy (crossover) Crossover: A total of 4 cycles of chemotherapy with each patient in each arm twice, crossover occurring after each cycle 1. Vomiting	1. T1 and T2: 17 out of 24 courses with prochlorperazine + placebo included vomiting vs. 16 out of 28 with THC + prochlorperazine, *p* = 0.31, n.s
Jones et al. (1982)	RCT, crossover	***n*** = 54; **Drop out: *n* = **30 **Cancer type:** Breast, lymphoma, ovary, lung, melanoma, testes, miscellanous	**Intervention:** Nabilone 2 mg **Comparison:** Placebo **Route of administration:** Orally **Regime:** Evening before chemotherapy, morning before chemotherapy, every 12 h following chemotherapy for at least 24 h, on various days corresponding to chemotherapy	T0: Baseline T1: Cycle 1 T2: Cycle 2 Crossover: after one cycle of chemotherapy 1. Nausea (rating: none = 0, mild = 1, moderate = 2, severe = 3) 2. Vomiting	1. T1 and T2 mean severity: nabilone = 2.0, placebo = 2.8, *p* < 0.001, sign.; less nausea was reported by nabilone *n* = 15 (63%), compared to placebo *n* = 1 (4%), *p* < 0.001; undecided patients *n* = 8 2. T1 and T2 mean number of episodes: nabilone = 7.2, placebo = 18.8, *p* < 0.001, sign.; less vomiting reported by nabilone *n* = 19 (79%), placebo *n* = 3 (13%), *p* < 0.001, sign.; undecided patients *n* = 2
Ungerleider et al. (1982)	RCT, crossover	***n*** = 214; **Drop out: *n* = **75 **Cancer type:** wide variety including carcinoma, sarcoma, lymphoma/Hodgkins, leukemia	**Intervention:** Delta 9-tetrahydrocannabinol (THC), body surface area < 1.4 m^2^ = 7.5 mg, body surface area 1.4 m^2^−1.8 m^2^ = 10 m mg, body surface area > 1.8 m^2^ = 12.5 mg **Comparison:** Prochlorperazine 10 mg **Route of administration:** Orally **Regime:** Two chemotherapy cycles were compared	T0: Baseline T1: Cycle 1 T2: Cycle 2 Crossover: after one cycle of chemotherapy 1. Nausea and vomiting with a homemade score (0–6)	1. T1 and T2: no significant difference between THC and prochlorperazine, no *p* value reported; subgroups of different chemotherapy regimens show no significant difference, no *p* value reported
Johannson et al. (1982)	RCT, crossover	***n*** = 27; **Drop out: *n* = **9 **Cancer type:** Cervix, fallopian tubes, ovary, testis, head and neck, bronchus, histiocytoma, fibrosarcoma, oligodendrioma, lymphoma	**Intervention:** Nabilone 2-mg capsules **Comparison:** Prochlorperazine 10-mg capsules **Route of administration:** Orally **Regime:** administration: 1 capsule every 12 h, up to 4, two cycles	T0: Baseline T1: Cycle 1 (1 day) T2: Cycle 2 (1 day) Crossover: after one cycle of chemotherapy 1. Severity of nausea with a questionnaire 2. Mean vomiting episodes 3. Number of vomiting episodes 4. Mean therapeutic effect with a homemade score (0–5)	1. No nausea: nabilone *n* = 3 (17%), prochlorperazine *n* = 0 (0%), mild: nabilone *n* = 6 (33%), prochlorperazine *n* = 3 (17%), moderate: nabilone *n* = 7 (39%), prochlorperazine *n* = 11 (61%), severe: nabilone *n* = 2 (11%), prochlorperazine *n* = 4 (22%); lower with nabilone compared to prochlorperazine, *p* = 0.027, sign 2. Ejection: nabilone 11.3, prochlorperazine 23.7, (*p* < 0.001); dry retching: nabilone 7.1; prochlorperazine 15.0, *p* < 0.01; total: nabilone 18.4, prochlorperazine 38.7, *p* < 0.001 3. 0 episodes: nabilone *n* = 3 (17%), prochlorperazine *n* = 0; 1–5 episodes: nabilone *n* = 3 (17%), prochlorperazine *n* = 2; 6–10 episodes: nabilone *n* = 5, prochlorperazine 2; 11–20 episodes: nabilone *n* = 4, prochlorperazine *n* = 5; > 20 episodes: nabilone *n* = 3 (17%), prochlorperazine *n* = 9 (50%), no *p* values reported 4. Nabilone more effective than prochlorperazine, nabilone: 2.4, prochlorperazine: 3.6; *p* < 0.00, sign
Levitt et al. (1982)	RCT, crossover	***n*** = 58; **Drop out: *n* = 20** **Cancer type:** Lung, ovarian, breast, others	**Intervention:** Nabilone 2 mg **Comparison:** Placebo **Route of administration:** Orally **Regime:** In the evening before chemotherapy, in the morning before chemotherapy, before going to bed on the day of chemotherapy, then twice daily	T0: Baseline T1: Cycle 1 T2: Cycle 2 (crossover) Crossover: after one cycle of chemotherapy 1. Effectiveness (measured as less nausea and less vomiting) 2. Number of vomiting episodes and severity of nausea (nausea: 0 = none, 1 = mild, 2 = moderate, 3 = severe)	1. T2: less vomiting with nabilone *n* = 29 vs. placebo *n* = 4, *p* < 0.001, sign., no difference in *n* = 3; less nausea with nabilone *n* = 26 vs. placebo *n* = 2, *p* < 0.001, sign., no difference in *n* = 8 2. T2: vomiting (mean): nabilone 2.97, placebo 7.47, *p* < 0.001, sign.; nausea (mean): nabilone 1.03; placebo 2.25, *p* < 0.001, sign
Wada et al. (1982)	RCT, crossover	***n*** = 114; **Drop out: *n* = **30 **Cancer type:** Lung, breast, ovarian, lymphoma, clonic, prostatic, adenocarcinoma, bladder, melanoma, pancreatic, esophagus, stomach, sarcoma, testis, others	**Intervention:** Nabilone 2 mg **Comparison:** Placebo **Route of administration:** Orally **Regime:** 8:00 PM the day before chemotherapy, 8:00 AM on the day of chemotherapy, then every 12 h after the last chemotherapy	T0: Baseline T1: Cycle 1 T2: Cycle 2, crossover Crossover: after one cycle of chemotherapy 1. **Effectiveness** (Nausea scale: 0 (none)–3 (severe), vomiting: count) 2. **Effectiveness**: less vomiting or nausea	1. Number of vomiting episodes per day (mean): nabilone 4.19, placebo 7.08, *p* < 0.001, sign.; severity of nausea: nabilone 1.22, placebo 1.96, *p* < 0.001, sign 2. Patients with less vomiting: nabilone *n* = 53 (58%), placebo *n* = 21 (23%), no difference *n* = 18 (19%), no *p* value reported; patients with less nausea: nabilone *n* = 56 (61%), placebo *n* = 9 (10%), no difference *n* = 27 (29%), no *p* value reported
Niedhart et al. (1981)	RCT, crossover	***n*** = 77; **Drop out: *n* = **50 **Cancer type:** Not given	**Intervention:** THC, 10 mg **Comparison:** Haloperidol, 2 mg **Route of administration:** Orally **Regime:** 2 h and 30 min before chemotherapy, additional doses started 1 h after chemotherapy and were then given at 3–4-h intervals up to a maximum of 8 doses	T0: Baseline T1: Before each dose, patients complete a questionnaire about vomiting and side effects T2: After 4 rounds Crossover: The precise timing of the crossover is not reported 1. Effectiveness (nausea and vomiting)	1. Effective in prevention of nausea and vomiting: THC 41%, haloperidol 37%, n.s.; Vomiting episodes: THC 9.9, haloperidol 13.2,
Einhorn et al. (1981)	RCT, crossover	***n*** = 100; **Drop out: *n* = **20 **Cancer type:** Sarcoma, Hodgkins’s disease, lymphoma, bladder, testicular	**Intervention:** Nabilone 2 mg **Comparison:** Prochlorperazine 10 mg **Route of administration:** Orally **Regime:** The results were collected over 10 days, with 5 days allocated to each arm, administration: 30 min before chemotherapy, every 6 h as needed afterwards	T0: Baseline T1: Days 1–5 of one cycle T2: Days 1–5 of one cycle Crossover: After one cycle of chemotherapy 1. Nausea with a homemade score (0–3) 2. Vomiting (frequency)	1. T1 and T2: Day 1: nabilone: 1.96, day 2: 1.18, day 3: 0.86, day 4: 0.93, day 5: 0.93; prochlorperazine: day 1: 2.21, day 2: 1.78, day 3: 1.53, day 4: 1.48, day 5: 1.38; nabilone vs. prochlorperazine overall: *p* < 0.001, day 1: *p* = 0.049, day 2: *p* < 0,001, day 3: *p* < 0.001, day 4: *p* = 0.001, day 5: *p* = 0.003, sign.; authors report significant shorter nausea in nabilone vs. prochlorperazine, no values given 2. T1 and T2: nabilone mean day 1: 7.05, day 2: 2.05, day 3: 01.08, day 4: 1.25, day 5: 1.12; prochlorperazine mean: day 1: 10.30, day 2: 5.09, day 3: 3.80, day 4: 3.24, day 5: 2.97; nabilone vs. prochlorperazine: day 1: *p* < 0.001, day 2: *p* < 0.001, day 3: *p* < 0.001, day 4: *p* = 0.001, day 5: 0.003; sign.; patients in the intervention group reported a 33% reduction in vomiting, no *p* value reported
Chang et al. (1981)	RCT, crossover	***n*** = 9; **Drop out: *n* = 1** **Cancer type:** Soft tissue sarcoma	**Intervention:** THC, 10 mg/m^2^, in case of vomiting approximately 17.4 mg as cigarette **Comparison:** Placebo **Route of administration:** Orally **Regime:** Every 3 h until 5 doses are reached	T0: Baseline T1: Data were collected every round from 7 am to midnight on the day of chemotherapy (one pared trail, THC-placebo or placebo-THC T2: Crossover Crossover: the exact crossover time cannot be classified 1. Nausea and vomiting with a questionnaire	1. Complete number of **v**omiting and gagging episodes (after crossover across all cycles): patient 1: THC 29, placebo 26, patient 2: THC 25, placebo 22, patient 3: THC 202, placebo 152, patient 4: THC 36, placebo 31, patient 5: THC 42, placebo 89, patient 6: THC 47, placebo 197, patient 7: THC 57, placebo 44, patient 8: THC 26, placebo 52, no *p* value reported, n.s.; total volume of vomit (ml):** p**atient 1: THC 1050, placebo 1750, patient 2: THC 3755, placebo 3950, patient 3: THC 3875, placebo 2950, patient 4: THC 680, placebo 1775, patient 5: THC 3650, placebo 6060, patient 6: THC 3275, placebo 4065, patient 7: THC 825, placebo 1170, patient 8: THC 755, placebo 455, no p value reported, n.s.; total nausea:** p**atient 1: THC 35, placebo 59, patient 2: THC 26, placebo 16, patient 3: THC 76, placebo 62, patient 4: THC 10, placebo 37, patient 5: THC 36, placebo 45, patient 6: THC 39, placebo 43, patient 7: THC 20, placebo 12, patient 8: THC 13, placebo 12, no *p* value reported, n.s.; total duration of nausea (minutes): patient**p**a 1: THC 530, placebo 772, patient 2: THC 122, placebo 37, patient 3: THC 1268, placebo 1165, patient 4: THC 135, placebo 372, patient 5: THC 373, placebo 495, patient 6: THC 850, placebo 875, patient 7: THC 210, placebo 72, patient 8: THC 95, placebo 8, no *p* values, n.s
Orr et al. (1981)	RCT, crossover	***n*** = 79; **Drop out: *n* = **24 **Cancer type:** Not given	**Intervention:** THC, 7 mg/m^2^ **Comparison:** Prochlorperazine, 7 mg/m^2^ **Comparison:** Placebo **Route of administration:** Orally **Regime:** Every 4 h for 4 doses, starting 1 h before chemotherapy	T0: Baseline T1: Cycle 1 T2: Cycle 2 (crossover) T3: Cycle 3 (crossover) Crossover: the exact crossover time cannot be classified 1. Nausea (2 questionnaires: telephone follow-up, patient questionnaire, 0–3 scale: 0 = no nausea, + 1 = present but not disruptive, + 2 = nausea affecting activity, + 3 = vomiting) 2. Correlation of nausea episodes with different chemotherapy regimens 3. Between-patient and within-patient comparisons 4. Episodes of nausea and vomiting (individual patient-specific)	1. Between group comparison: THC**:** no nausea *n* = 40, mild *n* = 7, severe = 5, vomiting = 3; prochlorperazine: no nausea *n* = 8, mild *n* = 1, severe *n* = 18, vomiting *n* = 18; placebo: no nausea *n* = 5, mild *n* = 8, severe *n* = 13, vomiting *n* = 29; no *p* values provided; total number of nausea episodes: THC 15, prochlorperazine 47, placebo 50 2. THC appears to have antiemetic prophylaxis for 5-fluorouracil, cyclophosphamide, and doxorubicin but not for nitrosoureas and nitrogen mustard; nausea episodes: doxorubicin: THC 4, prochlorperazine 10, placebo 10; cyclophosphamide: THC 0, prochlorperazine 3, placebo 4, doxorubicin-cyclophosphamide: THC 0, prochlorperazine 4, placebo 3; no *p* values reported 3. Within patient comparison: prochlorperazine was only slightly better than placebo, THC was better than prochlorperazine, no *p* value reported, no comment on significance; between-group comparison: THC was better than prochlorperazine, no *p* values provided, no comment on significance 4. Individual patient comparison: THC vs. prochlorperazine: no nausea in both THC and prochlorperazine: *n* = 4, nausea only in THC: *n* = 4, vomiting in THC and no nausea in prochlorperazine: *n* = 0, no nausea in THC but nausea in prochlorperazine: *n* = 24, nausea in both THC and prochlorperazine: *n* = 4, vomiting in THC and nausea in prochlorperazine: *n* = 1, no nausea in THC but vomiting in prochlorperazine: *n* = 12, nausea in THC and vomiting in prochlorperazine: *n* = 4, vomiting in both THC and prochlorperazine: *n* = 2, *p* < 0.001, sign.; THC vs. placebo: no nausea in both THC and placebo: *n* = 2, nausea only in THC: *n* = 2, vomiting in THC and no nausea in placebo: *n* = 1, no nausea in THC but nausea in placebo: *n* = 18, nausea in both THC and placebo: *n* = 3, vomiting in THC and nausea in placebo: *n* = 3, no nausea in THC but vomiting in placebo: *n* = 20, nausea in THC and vomiting in placebo: *n* = 7, vomiting in both THC and placebo: *n* = 2, *p* < 0.001, sign.; prochlorperazine vs. placebo: no nausea in both prochlorperazine and placebo: *n* = 1, nausea only in prochlorperazine: *n* = 4, vomiting in placebo and no nausea in prochlorperazine: *n* = 3, no nausea in placebo but nausea in prochlorperazine: *n* = 1, nausea in both prochlorperazine and placebo: *n* = 11, vomiting in placebo and nausea in prochlorperazine: *n* = 17, no nausea in placebo but vomiting in prochlorperazine: *n* = 3, nausea in placebo and vomiting in prochlorperazine: *n* = 6, vomiting in both prochlorperazine and placebo: *n* = 9, *p* = 0.05,n.s
Colles et al. (1980)	RCT, crossover	***n*** = 35; **Drop out: *n* = **0 **Cancer type:** Solid tumors	**Intervention:** THC, 4 mg per capsule, 1 capsule per 0.33 m^2^ body surface area **Comparison:** Thiethylperazine, 2.2 mg per capsule, 1 capsule per 0.33 m^2^ body surface area **Comparison:** Metoclopramide 5 mg **Route of administration:** Orally and intravenously **Regime:** 2 h before chemotherapy, 2 h after chemotherapy, and 6 h after chemotherapy + identical placebo administration	T0: Baseline T1: Cycles 1 and 2 T2: Cycles 3 and 4 T3: Cycles 5 and 6 Crossover: crossover after 2 cycles of chemotherapy 1. Nausea and vomiting (scoring system: 0 = no nausea and vomiting, 1 = some nausea but no vomiting, 2 = nausea and occasional vomiting, 3 = repeated nausea and vomiting, 4 = severe nausea and uncontrollable vomiting, 5 = additional medications required 2. Vomiting (0–6 h and 6–24 h after chemotherapy)	1. Results were compared after T3: The emetic effect (rated by patients and staff) was lower with metoclopramide vs. THC or thiethylperazine, no *p* value reported, n.s.; staff observation (mean): THC 0.86, thiethylperazine 0.92, metoclopramide 0.59, n.s., no *p* value; patient observation (mean): THC 0.69, thiethylperazine 0.57, metoclopramide 0.42, n.s., no *p* value 2. 0–6 h (mean): THC 2.70, thiethylperazine 2.39, metoclopramide 1.98; 6–24 h (mean): THC 1.80, thiethylperazine 1.74, metoclopramide 1.59; n.s., no *p* value reported
Sallan et al. (1980)	RCT, crossover	***n*** = 84; **Drop out: *n* = **11 **Cancer type:** Not given	**Intervention:** THC; 10 mg per m^2^ body surface area **Comparison:** Prochlorperazine, 10 mg **Route of administration:** Orally **Regime:** Each round consists of 3 doses, to be taken every 4 h, first dose 1 h before chemotherapy	T0: Baseline T1: Course 1 (1 day) T2: Course 2 (1 day) T3: Course 3 (1 day) Crossover: The exact crossover time cannot be classified 1. CR = complete response (no nausea and vomiting), PR = partial response (reduction in nausea and vomiting), NR = no response (no reduction)	1. T1**:** for patients who completed round 1 (*n* = 27, 15 THC, 12 prochlorperazine): THC: CR *n* = 6, NR *n* = 9, prochlorperazine: CR *n* = 1, NR *n* = 1, *p* < 0.05, sign.; T3 (number of courses): THC**:** CR = 36, prochlorperazine:16, no PR: THC = 10, prochlorperazine = 15, NR: THC = 33, prochlorperazine = 47, no *p* values provided; high emetogenic subgroup (number of courses): THC: CR = 18, PR = 4, NR = 16 out of 38, prochlorperazine: CR = 7, PR = 9, NR = 20, total = 36, moderate emetogenic subgroup: THC: CR = 13, PR = 5, NR = 12, out of 30, prochlorperazine: CR = 8, PR = 4, NR = 20, total = 32; least emetogenic subgroup: = CR = 5, PR = 1, NR = 5 out of 11, prochlorperazine: CR = 1, PR = 2, NR = 7, total 10, no *p* values provided
Steel et al. (1980)	RCT, crossover	***n*** = 55; **Drop out: *n* = **18 **Cancer type:** Not given	**Intervention:** Nabilone, 2 mg **Comparison:** Prochlorperazine, 10 mg **Route of administration:** Orally **Regime:** Every 12 h for 3–4 doses, first dose given at night before chemotherapy	T0: Baseline T1: Intervention T2: Crossover Crossover: After 1 cycle of chemotherapy 1. Duration and subjective perception of nausea (Intensity scale: 0 = none, 1 = mild, 2 = moderate, 3 = severe) 2. Vomiting (duration)	1. Duration with nabilone: DDP > low-dose DDP > other chemotherapies; high-dose DDP**:** median duration: nabilone 1.79, prochlorperazine 1.17, range of duration: nabilone 0–3, prochlorperazine 0–2, intensity (mean): nabilone 1.33, prochlorperazine 1.60, no *p* values provided; low-dose DDP: median duration: nabilone 0.75, prochlorperazine 0.75, range of duration: nabilone 0–2, prochlorperazine 0–2, intensity (mean): nabilone 1.50, prochlorperazine 1.70, no *p* values provided; other chemotherapies: median duration: nabilone 0.62, prochlorperazine 1.05, range of duration: nabilone 0.5–2, prochlorperazine 0.5–3, intensity (mean): nabilone 1.82, prochlorperazine 2.36, no *p* values provided; Overall: median duration: nabilone 0.73, prochlorperazine 1.02, range of duration: nabilone 0–3, prochlorperazine 0–3, intensity (mean): nabilone 1.53, prochlorperazine 1.86, overall intensity and duration of nausea is lower with nabilone, n.s., no *p* value provided 2. High-dose DDP**:** median duration: nabilone 2.83, prochlorperazine 3.50, duration range (hours): nabilone 0–48, prochlorperazine 0–36, frequency (hours, median): nabilone 7, prochlorperazine 6, range: nabilone 0–35, prochlorperazine 0-continuous, no *p* values provided; low-dose DDP: median duration: nabilone 1.25, prochlorperazine 4.25, duration range (hours): nabilone 0–7, prochlorperazine 0–7, frequency (hours, median): nabilone 6.5, prochlorperazine 7.5, range: nabilone 0–34, prochlorperazine 0-continuous, no *p* values provided; other chemotherapies: median duration: nabilone 4.25, prochlorperazine 6.75, duration range (hours): nabilone 0.5–10, prochlorperazine 0–48, frequency (hours, median): nabilone 18, prochlorperazine 6, range: nabilone 2-continuous, prochlorperazine 0-continuous, no *p* values provided; overall: nabilone 3.19, prochlorperazine 5.17, duration range (hours): nabilone 0–48, prochlorperazine 0–36, frequency (hours, median): nabilone 6, prochlorperazine 11.5, range: nabilone 0-continuous, prochlorperazine 0-continuous, no *p* value reported
Herman et al. (1979)	RCT, crossover	***n*** = 152; **Drop out: *n* = **39 **Cancer type:** Various types of cancer, no detailed report	**Intervention:** Nabilone 1-mg capsules **Comparison:** Prochlorperazine 5-mg capsules **Route of administration:** Orally **Regime:** Two chemotherapy cycles were compared, 1 cycle comprises a maximum of 5 days, capsule administration: 2 capsules every 8 or 6 h	T0: Baseline T1: Cycle 1 (max. 5-day intervention) T2: Cycle 2 (crossover) Crossover: after 1 cycle of chemotherapy 1. Complete response (total absence of nausea and vomiting), partial response (reduction of ≤ 50%), no response (reduction > 50%) 2. Vomiting episodes and s	1. Complete response: nabilone *n* = 9 (8%), prochlorperazine *n* = 0 (0%), no *p* value reported, n.s.; partial response: nabilone *n* = 81 (72%), prochlorperazine *n* = 36 (32%), *p* < 0.01; no response: nabilone *n* = 36 (20%), prochlorperazine *n* = 77 (68%), no *p* value reported; within comparison: number of complete responses + partial responses is significantly better with nabilone, *p* < 0.001 2. Episode days 1–5: significantly fewer episodes with nabilone compared to prochlorperazine, *p* < 0.001; severity days 1–5 mean: significantly lower with nabilone, *p* value range < 0.05 to < 0.001
Chang et al. (1979)	RCT, crossover	***n*** = 15; **Drop out: *n* = 0** **Cancer type:** Osteogenic sarcoma	**Intervention:** THC, 10 mg/m^2^, in case of vomiting approximately 17.4 mg as cigarette **Comparison:** Placebo **Route of administration:** Orally **Regime:** Every 3 h until 5 doses are reached	T0: Baseline T1: 3 paired (6) trails in a randomized order Crossover: after each paired trail 1. **Nausea and vomiting** (with a questionnaire, Excellent Effect = > 80% reduction, Good Effect = between 80 and 30%, poor = less than 30% reduction in all 4 nausea and vomiting parameters with THC compared to placebo	1. Results of the 64 Completed Trials T1: reduction of nausea and vomiting THC: *n* = 14 out of *n* = 15 (*n* = 8 excellent effect, *n* = 6 good effect), no *p* value provided; analysis (Koch’s method) of the first crossover round: THC vs. placebo in terms of the number of vomiting and gagging episodes: *p* < 0.02, sign., intensity of nausea *p* < 0.01, sign.,duration of nausea *p* < 0.01, sign., and volume of vomit *p* < 0.01, sign.; **f**irst crossover round: THC is significantly better than group placebo in reducing nausea, *p* < 0.01, sign.; second crossover round: THC is not significantly better than placebo in reducing nausea, no *p* value reported; T1: two additional analysis methods: Difference between paired trials THC and placebo, as well as the blocked Wilcoxon test: THC was significantly better than placebo in terms of the number of vomiting and gagging episodes, nausea, duration of nausea, and volume of vomit, no mean values reported, *p* < 0.001
Kluin-Neleman et al. (1979)	RCT, crossover	***n*** = 11; **Drop out: *n* = **2 **Cancer type:** Hodgkin or nonHodgkin lymphoma	**Intervention:** THC, 5 mg **Comparison:** Placebo **Route of administration:** Orally **Regime:** 2 capsules 2 h before chemotherapy, 2 capsules 4 and 8 h after chemotherapy	T0: Baseline T1: Cycle 1 (days 1and 8) T2: Cycle 2 (days 1 and 8) Crossover: after 1 cycle of chemotherapy 1. Nausea and vomiting (measured with a scale 1–5: 1 = no improvement, 2 = slightly better, 3 = clear improvement but still nausea and vomiting, 4 A = no vomiting but nausea, 4B = no nausea but vomiting, 5 = no nausea and no vomiting)	1. Mean score comparing day 1: T1: THC: 2.27 ± 1.03, placebo: 1.09 ± 0.30, *p* < 0.01, sign., mean score comparing day 8: THC: 3.93 ± 1.33, placebo: 1.67 ± 0.89, *p* < 0.01, sign
Frytak et al. (1997)	RCT	***n*** = 117; **Drop out: *n* = **19 **Cancer type:** Colorectal, gastric liver, miscellaneous	**Intervention:** Delta-9-tetrahydrocannabinol (THC) **Comparison:** Prochlorperazine 10 mg **Comparison:** Placebo **Route of administration:** Orally **Regime:** Administered three times daily for 5 days, the results were collected over 4 days	T0: Baseline T1: Day 1 (stronger chemotherapy than on days 2–4) T2: Day 2 T3: Day 3 T4: Day 4 1. Nausea and vomiting (no nausea unless asked, nausea: vomiting only once in 24 h, vomiting: 2 times or more often)	1. Day 1: no nausea and vomiting (%): THC 42, prochlorperazine 42, placebo 19; nausea only (%): THC 5, prochlorperazine 2, placebo 16; nausea and vomiting (%)**:** THC 53, prochlorperazine 56, placebo 65; placebo vs. THC and prochlorperazine sign. no p value reported, THC vs. prochlorperazine, *p* = 0.05, n.s.; days 2–4: no nausea and vomiting (%): THC 57, prochlorperazine 72, placebo 53; nausea only (%): THC 21, prochlorperazine 14, placebo 29; vomiting (%): THC 21, prochlorperazine 14, placebo 18; THC vs. prochlorperazine *p* = 0.02, n.s
Sallan et al. (1975)	RCT, crossover	***n*** = 22; **Drop out: *n* = **12 **Cancer type:** Not given	**Intervention:** THC; 15 mg per m^2^ body surface area **Comparison:** Placebo **Route of administration:** Orally **Regime:** 2 h before chemotherapy, 2 and 6 h after, 1 day, crossover	T0: Baseline T1: Day 1 T2: Day 2—crossover T3: Day 3—crossover Crossover: After each day 1. Vomiting (CR = no vomiting, PR = at least 50% reduction, NR = THC: no reduction or less than 50% reduction	1. 29 cycles were evaluated, THC: 15 and placebo: 14, *n* = 10, NR in placebo: all cycles, CR in THC: 5 cycles, PR in THC: 7 cycles, NR in THC: 3 cycles, THC vs. placebo, *p* < 0.001, sign

### Excluded studies

Twenty RCTs were excluded because they did not meet the criteria for relevant outcomes (17 RCTs), publications (two RCTs), or studies (one RCT). A list of all excluded studies can be found in Table S2.

### Risk of bias in the included studies

**Fig. 2:**
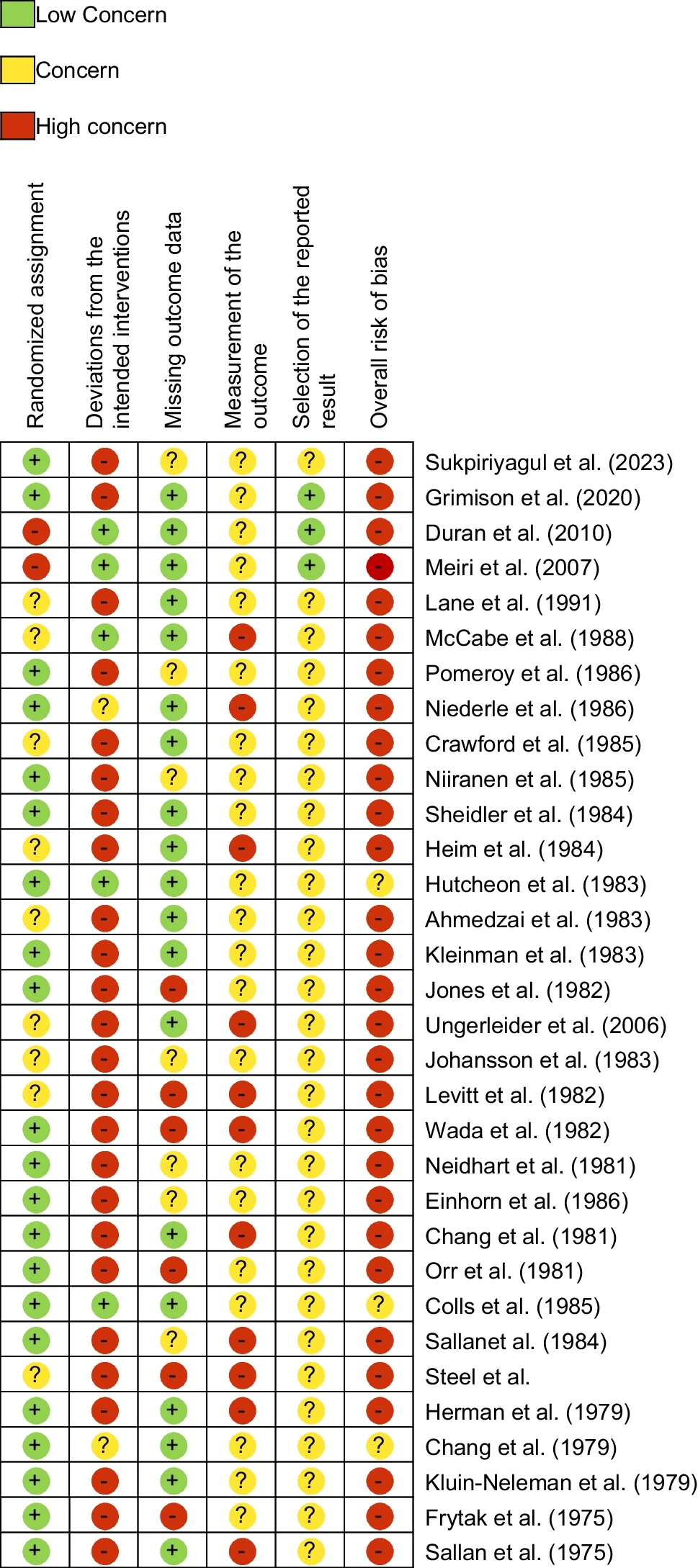
ROB figure

The methodical quality was assessed with the Cochrane revised Risk of Bias Tool 2.0 [7]. The results are presented in Table S3 and Fig. 2. None of the included studies had a high quality, three were of moderate quality [19, 31, 35], and 29 had a poor [10–18, 20–30, 32–34, 36–41] quality. Many studies presented a high risk of bias, primarily due to concerns regarding blinding, typically arising from the well-known side effects of cannabis. ITT analysis was conducted in only two RCTs [39, 41], and power analysis was conducted in only four RCTs [10, 17, 39, 40]. Furthermore, no control for multiple testing was used in two RCTs [10, 39], and comparability of baseline values was frequently uncertain, with limited information provided in 14 RCTs [11, 13, 15, 17, 20, 28, 30, 32–35, 38, 39, 41]. Even when studies incorporated blinding, potential unblinding occurred due to known adverse effects during the course of the study, which largely led to a high risk of bias in the majority of these studies [10–18, 20–27, 29, 30, 32, 33, 36–41].

## Cannabinoids as part of antiemetic therapy

### Cannabinoids added to antiemesis according to modern standards (moderate to highly emetogenic chemotherapy)

Nausea and vomiting were assessed in one RCT that used antiemesis agents following modern standards for CINV [10]. In this trial, cannabinoids were added to standard antiemetics (5-HT3 receptor antagonists, NK1 receptor antagonists, and a steroid) and compared with a placebo. Although the methodology distinguished between acute and delayed CINV, the results only reflect a period of 0–120 h for both types of CINV.

In this crossover study, patients were assigned to daily THC/CBD or placebo for 5 days per cycle following chemotherapy. After two cycles, the THC/CBD group had significantly more complete responses (no vomiting, no rescue medication) within the 0–120-h period (*p* = 0.041) [10]. However, these findings do not account for any delayed effects that may occur beyond the 0–120-h interval. Additionally, self-reported complete responses (no vomiting, nausea < 2 on a numeric rating scale, no rescue medication) were 25% in the THC/CBD group versus 14% in the placebo group, demonstrating a significant advantage for THC/CBD (*p* = 0.04) [10].

## Antiemesis to outdated standards

Thirty-one RCTs assessing nausea and/or vomiting using antiemetic treatment did not use modern standards [11–41]. Most studies have used a crossover design, with six exceptions utilizing a parallel group design [11, 13, 17, 37, 39, 41]. Eight RCTs prohibited other antiemetics [14, 16, 18, 22, 23, 25, 28, 37], one allowed them but did not specify them [17]; in three RCTs, background antiemetic prophylaxis was provided to traditional standards [39–41], and no additional information was provided in the remaining studies. The studies predominantly focused on a 24-h administration window targeting acute CINV, with the exception of one study that additionally addressed anticipatory CINV [11] and three studies that considered both acute and delayed CINV [39–41].

### High emetogenic chemotherapy

Eight RCTs compared prochlorperazine to cannabinoids. One study reported that THC was significantly more effective than prochlorperazine for nausea over three chemotherapy cycles (*p* < 0.001) [30]. Six RCTs [16, 20, 24, 28, 33, 34] favored or found nabilone to be equally effective for acute CINV, with significant or equal reductions in nausea and/or vomiting: reduced nausea severity (*p* = 0.027) and vomiting episodes (*p* < 0.001) over two 1-day chemotherapy schedules [24]; a superior effect for nausea and vomiting after two cycles (*p* < 0.001 to *p* = 0.049) [28]; decreased nausea and vomiting over 5 days (*p* < 0.05 to *p* < 0.001) [34]; and fewer vomiting episodes (*p* < 0.05) but no significant difference in overall nausea severity in another study [16].

An additional study [20] reported significant differences in nausea on days 1 to 3, retching on days 1 and 2, and vomiting on days 2 and 3 (*p* < 0.001 to *p* < 0.05). Two studies reported no statistically significant differences between nabilone (no *p* value reported) [33] or levonantradol (*p* = 0.6) [17] compared with prochlorperazine in reducing CINV after two chemotherapy cycles. Five RCTs compared cannabinoids to other antiemetics. Nabilone was significantly more effective than alizaprid (nausea and vomiting, *p* < 0.01) [14] and domperidone regarding vomiting (*p* < 0.01) but not for nausea (*p* > 0.05) [13] after two cycles. Levonantradol was superior to metoclopramide when comparing two cycles (*p* < 0.05) [18] and chlorpromazine for vomiting (*p* < 0.05), with similar efficacy for nausea (no *p* value reported) after one cycle [19].

One study reported no significant differences between nabilone and metoclopramide in terms of nausea (*p* > 0.05) or vomiting (*p* > 0.1) after four cycles [15]. Three RCTs compared cannabinoids to placebo for two cycles: one reported that THC was superior for nausea and vomiting on days 1 and 8 (*p* < 0.01) [36]; one reported that nabilone was superior for vomiting (*p* < 0.001) and nausea (*p* < 0.001) [22]; and another reported no significant effect for THC after two cycles, with *p* values unreported [29].

### Moderate to high emetogenic chemotherapy

The most recent published study revealed that THC oil significantly outperformed placebo over 5 days (0–120 h, *p* = 0.001) and in the acute period (0–24 h, *p* = 0.001), with no significant differences in terms of nausea occurring on day 3 (*p* = 0.097) [40]. In another study [39], dronabinol, ondansetron, and their combination were compared to placebo for CINV over 5 days. On day 1 after chemotherapy (acute period), only the combination was compared with the placebo and showed significantly better protection (*p* = 0.024, post hoc analysis) in terms of a complete response, including vomiting, nausea, and no need for rescue medication [39].

In the delayed period for complete response (days 2 to 5 after chemotherapy), exploratory analysis revealed a significant benefit of ondansetron over placebo (*p* = 0.04) [39] and a clinically relevant improvement for all active treatments compared with placebo, although no *p* value was reported for the latter comparison. When nausea and vomiting were examined separately from days 2 to 5, all the active treatments significantly reduced nausea compared with the placebo (*p* < 0.05), but on average no significant differences in vomiting episodes were observed [39].

In a small study, THC achieved a 25% complete response (i.e., no nausea or vomiting) after four 1-day courses (24-h intervals), while prochlorperazine had 0%; *p* values were not reported [12].

### Moderate emetogenic chemotherapy

In one study, a THC/CBD spray (Sativex®) was compared with a placebo for 4 days after chemotherapy. The intervention resulted in a significantly better complete response for vomiting and nausea during the overall (0–120 h) and delayed (24–120 h) periods, but not in the acute period, with no *p* value provided [41].

### Moderate and low emetogenic chemotherapies

One trial revealed no significant difference between THC and prochlorperazine for nausea and vomiting over 4 days (*p* = 0.05 to *p* = 0.22) [37]. Another study reported that THC was significantly more effective than placebo in reducing vomiting and nausea during six chemotherapy cycles (*p* < 0.001) [35].

### Chemotherapy across different emetogenic groups

Four RCTs compared a cannabinoid to prochlorperazine and reported that THC was significantly better for a 1-day course (complete response, no *p* value provided) [32], no difference in vomiting after four cycles with THC (*p* = 0.31) [21], no significant difference after two cycles for CINV with THC [23], and no difference in nausea and vomiting for acute CINV after 5 days of chemotherapy [11]. Two RCTs reported no significant differences between THC and other antiemetics (haloperidol and thiethylperazine, metoclopramide) for nausea and vomiting, after four chemotherapy cycles [27] or within 24 h [31], with no *p* values reported. Three RCTs comparing cannabinoids to placebo showed that THC significantly reduced vomiting (*p* < 0.001) [38], and nabilone was superior to placebo for nausea severity and vomiting episodes after two cycles (*p* < 0.001) [25, 26].

Only one trial examined anticipatory nausea and reported that prochlorperazine was more effective than dronabinol in reducing nausea and vomiting (*p* < 0.05) [11].

## Side effects related to cannabis-based therapy

One study that used modern antiemetic standards reported that cannabinoid-related side effects were more common with cannabinoids (31% vs. 7%, *p* = 0.002), such as sedation (19% vs. 4%, *p* = 0.002) and dizziness (10% vs. 1%, *p* = 0.03) [10]. Studies with traditional antiemetic standards reported no significant differences in sedation and dizziness (*p* = 0.064) [40]. The majority of the remaining studies reported cannabis-related side effects, with sedation, somnolence, and a dry mouth being the most common [11–38]. In one trial, cannabinoids had significantly more side effects than other antiemetics did (*p* < 0.01) [11], and an overall higher dropout rate was associated with cannabinoids. This increased dropout rate associated with cannabinoid use is partly attributed to such adverse effects as dysphoria [23, 26, 30].

## Discussion

This systematic review offers a comprehensive analysis of RCTs investigating the efficacy of cannabinoids in mitigating CINV and other cancer treatment–related nausea and vomiting. The analysis revealed that cannabinoids could be an effective treatment option against nausea and vomiting associated with moderate and highly emetogenic chemotherapy compared to low or unclassified emetogenic chemotherapy, across all included studies. All three included randomized controlled trials conducted in the context of moderately to highly emetogenic chemotherapy, involving at least one of the newer antiemetics and differentiating between acute and delayed CINV, yielded statistically significant outcomes favoring cannabinoids over placebo [39–41]. The studies had large sample sizes and adequate power [40], whereas others included small samples or were underpowered [39, 41] and had a high [39–41] risk of bias. Compared to placebo, all studies involving chemotherapy found cannabinoids effective, although one study [29] with low THC levels lacked significance. Nevertheless, those studies showed moderate [35] or high [22, 25, 26, 29, 36–41] risk of bias, noting issues such as study discontinuation and patient dropout due to serious vomiting and/or nausea and overall side effects [22, 25, 26, 36, 37]. One study with high risk of bias included both a new antiemetic (ondansetron) and a cannabinoid compared with a placebo [39]. In this study, dronabinol and ondansetron were similarly effective, and their combination was significantly better than placebo for acute CINV. However, baseline risk factors were imbalanced in favor of the dronabinol arm. Specifically, the dronabinol group included older patients, fewer women, and a higher proportion of chemotherapy-naïve individuals compared to the placebo arm. Additionally, the dronabinol arm had fewer breast cancer patients, implying that fewer participants received AC chemotherapy (a highly emetogenic regimen) in this group. Although the study initially planned to enroll 464 patients, only 64 were randomized, with 61 (95%) included in the intention-to-treat (ITT) population and just 51 (80%) completing the trial. In light of these significant methodological limitations and imbalances, the study appears to be severely biased. Therefore, no reliable conclusions can be drawn from its findings.

Overall, the extensive variation in administration forms, dosages, durations, and patient questionnaires employed in the included studies complicates a cohesive summary of the findings. The majority of studies investigating CINV in refractory patients have used outdated comparator agents, most commonly prochlorperazine, which is no longer considered standard of care. A critical methodological concern is that patients were often deemed refractory to the same antiemetic used as the comparator in the subsequent treatment cycle. It is therefore unsurprising that patients demonstrated improved outcomes when switched to an alternative agent, as continued use of a previously ineffective antiemetic would be expected to yield poor results. This design flaw substantially limits the validity of the conclusions drawn from such studies. In addition, the vast majority of studies fail to adequately report on patient dropouts, leading to a likely underestimation of adverse events associated with cannabinoid use. Furthermore, the average age of participants in most studies is significantly lower than that of the typical patient population receiving chemotherapy in clinical practice. Given the known differences in pharmacodynamics and susceptibility to adverse effects between younger and older adults, a focused evaluation of cannabinoid-related adverse events in older patients is warranted and would provide important clinical insight.

Since the late 1980 s, new antiemetics, such as ondansetron, and NK1 antagonists, such as aprepitant, have been introduced and have largely replaced prochlorperazine due to improved tolerance [42]. According to the 2023 guidelines of the American Society of Clinical Oncology (ASCO), first-line prophylaxis for highly emetogenic chemotherapy typically includes a three-drug regimen consisting of a 5-HT3 receptor antagonist, an NK1 receptor antagonist, and dexamethasone, with the optional addition of olanzapine [43]. The guidelines of the National Comprehensive Cancer Network (NCCN) align with this approach, offering similar drug combinations and stratifying treatment according to the emetogenic potential of the chemotherapy regimen [44]. Notably, cannabinoids are not recommended as first- or second-line therapies in either guideline, but are mentioned as a last-resort or palliative option, generally reserved for patients who do not respond adequately to conventional antiemetics.

This systematic review included four RCTs involving at least one of the newer antiemetic medications for prophylaxis [10, 39–41]; however, only one study utilized a comprehensive background prophylaxis for CINV, including all three drug classes, in accordance with current standards [10]. This study demonstrated that cannabinoids were significantly more effective than placebo [10]. The study lacked clarity in endpoint timing and used individualized dosing without subgroup analyses. The heterogeneous cancer types and chemotherapy regimens limit the applicability of the findings. Moreover, the study was substantially underpowered. It employed a significance level of 10% (reporting 90% confidence intervals rather than the conventional 95%) and was designed to detect only large effect sizes, specifically a 20% difference. As the observed differences were smaller than this threshold, the study lacks the statistical power to support any definitive conclusions. The study exhibited a high risk of bias [10] and future research with more defined populations is needed to assess the use of cannabinoids according to current standards. After the conclusion our literature search, another study was published comparing the efficacy of cannabinoids against CINV with a guideline-conform therapy consisting of a corticosteroid and 5-hydroxytryptamine antagonist, a neurokinin-1 antagonist, and olanzapine [45]. In patients receiving moderately or highly emetogenic chemotherapy, oral THC:CBD significantly improved control of nausea and vomiting despite standard antiemetic therapy, increasing complete response rates from 8 to 24%. However, its use was associated with more frequent mild to moderate side effects, such as sedation and dizziness.

Concerning studies with traditional prophylactic standards, most studies comparing cannabinoids to prochlorperazine and other antiemetics for nausea and vomiting have shown cannabis to be superior [13, 14, 16, 18–20, 24, 28, 30, 32, 34] or equally effective [11, 13, 15–17, 21, 23, 27, 31, 33, 37] for treating nausea and/or vomiting, with most of the chemotherapies being moderate or highly emetogenic.

Nevertheless, most studies had moderate to high risks of bias. One study revealed that prochlorperazine was more effective for anticipatory nausea [11], but it had significant methodological limitations. More importantly, anticipatory nausea follows a different mechanism than CINV and is currently treated primarily with antipsychotics and benzodiazepines, as it arises from a conditioned physiological response.

All studies included in our review used psychoactive cannabinoids, and all studies involved medical cannabis, which is governed by strict controls in its composition. However, the 2018 UN report suggested that daily medical cannabis use could result in dependence, with a risk estimated at up to one in three users, particularly for those using THC daily for chronic pain compared with weekly use for chemotherapy-induced nausea [46]. Both psychological and physical dependence must be considered when cannabis-based medications are used. Moreover, the regular use of marijuana of the patients receiving cannabinoids against CINV must be taken into account. For example, one study [35] was conducted in the context of high-dose methotrexate (MTX), a treatment regimen that is now rarely used. It compared inhaled marijuana with placebo and included only 15 patients, 11 of whom were regular marijuana users. A placebo-controlled trial in the setting of high-dose MTX would not meet current ethical standards and would likely be considered unethical today.

In the context of potential harms of cannabinoid therapy, the MASCC (Multinational Association of Supportive Care in Cancer) guidelines state that most side effects of cannabinoids appear to be mild to moderate, but some patients discontinued treatment due to side effects [47]. Common side effects include dry mouth, dizziness, and somnolence, which increase the risk of accidents, especially for the elderly. In addition, cannabinoids may cause serious side effects such as dysphorie, hallucinations, and dyspnea. Several studies have reported a sensation of a “high” [12, 20, 23, 28–30, 33, 35, 37, 38], with significance in favor of cannabinoids reported in two RCTs [28, 37]. Two RCTs reported a correlation between the sensation of a “high” and the antiemetic effects of cannabis (*p* < 0.001 [32]) [30], suggesting that side effects may indicate a therapeutic response. Furthermore, individual variability in the sensation of a “high” could influence the medication’s efficacy.

## Limitations of review

This review has some limitations that must be considered in the context of its applicability to the clinical practice. First, it should be noted that only studies published in English or German until June 2024 and those with an RCT design were included. Additionally, the considerable heterogeneity among the studies must be recognized and made their comparability challenging. Furthermore, owing to the highly varied data, especially in terms of patient characteristics, dosage, and the use of different self-made questionnaires, a meta-analysis on CIPN was not included.

## Conclusion

While the findings of this review support the potential efficacy of cannabinoids, they also underscore that the overall quality of the evidence remains low. Due to the heterogeneity in chemotherapy protocols and cannabinoid formulations among the studies, definitive conclusions on the efficacy of cannabinoids against CINV and their broader clinical applicability may not be drawn at this point, and studies employing rigorous methodologies are necessary to conclusively evaluate the efficacy of cannabinoids against CINV. Given that only one study with a high risk of bias meets current antiemetic standards and considering the potential for adverse effects, the clinical evidence supporting the use of cannabinoids for the management of chemotherapy-induced nausea and vomiting (CINV) remains insufficient. If a cannabinoid is to be considered for this indication, it should only be used after guideline-recommended antiemetic therapies have proven ineffective.

## Supplementary Information

Below is the link to the electronic supplementary material.

Supplementary file1 (DOCX 48 KB)

## Data Availability

No datasets were generated or analysed during the current study.

## Abbreviations

5-HT3: 5-Hydroxytryptamine-3
ASCO: American Society of Clinical Oncology
CBD: Cannabidiol
CINV: Chemotherapy-induced nausea and vomiting
ITT: Intention-to-treat
MASCC: Multinational Association of Supportive Care in Cancer
MTX: Methotrexate
NCCN: National Comprehensive Cancer Network
NK1: Neurokinin-1
RCT: Randomized controlled trial
THC: Delta-9-tetrahydrocannabinol
AC: Adriamycin, cyclophosphamide
