# Supplementary material for: Cannabinoids for the prevention of chemotherapy-induced nausea and vomiting in oncological therapy: a systematic review
Source: J Cancer Surviv. 2025 Aug 12;20(Suppl 1):1296–317. doi: 10.1007/s11764-025-01876-4 (PMC13676573; doi:10.1007/s11764-025-01876-4)
Supplement: Supplementary file 1 [file 11764_2025_1876_MOESM1_ESM.docx]

**Supplementary Information**

**Cannabinoids for the prevention of chemotherapy-induced nausea and vomiting in oncological therapy: A systematic review**

**Authors:**

Sarah F. Kemmner, Klinik für Innere Medizin II, Hämatologie und Internistische Onkologie, Universitätsklinikum Jena, Am Klinikum 1, 07747 Jena, Germany, ORCID: 0009-0000-3227-4549

Jennifer Dörfler, MSc., Klinik für Innere Medizin II, Hämatologie und Internistische Onkologie, Universitätsklinikum Jena, Am Klinikum 1, 07747 Jena, Germany,

Prof. Dr. med. Jutta Huebner, Klinik für Innere Medizin II, Hämatologie und Internistische Onkologie, Universitätsklinikum Jena, Am Klinikum 1, 07747 Jena, Germany

**Contact:**

Prof. Dr. med. Jutta Huebner, Klinik für Innere Medizin II, Hämatologie und Internistische Onkologie, Universitätsklinikum Jena, Am Klinikum 1, 07747 Jena, Germany, Tel.: 0049-3641-9324256, Fax: 03641 9324217, E-Mail: jutta.huebner@med.uni-jena.de; E-Mail: Jenifer.Doerfler@med.uni-jena.de

**Corresponding author:**

Sarah F. Kemmner, Klinik für Innere Medizin II, Hämatologie und Internistische Onkologie, Universitätsklinikum Jena, Am Klinikum 1, 07747 Jena, Germany, Tel.: 01727461430, E-Mail: sarah@kemmner.com

Journal of Cancer Survivorship

**Table S1: Search strategy**

| Database | Search strategy |
| --- | --- |
| Ovid Medline | **1** exp Cannabinoids OR exp Medical Marijuana OR cannabis.mp OR cannabinoid$.mp OR cannabidiol.mp OR cannabinol.mp OR Mariuana.mp OR Marihuana.mp OR Marijuana.mp OR hemp.mp OR hanf.mp OR phytocannabinoid.mp OR tetrahydrocannabinol.mp OR THC.mp OR CBD.mp OR tetrahydrocannabivarin.mp.  **2** nabiximols.mp OR sativex.mp OR dronabinol.mp OR marinol.mp OR namisol.mp OR nabilon.mp  **3** (synthetic adj1 cannabi$).mp OR (synthetic adj1 marijuana).mp OR (synthetic adj1 marihuana).mp OR K2.mp OR spice.mp  **4** exp neoplasms/or neoplasm$.mp or cancer$.mp. or tumo?r$.mp. or malignan$.mp. or oncolog$.mp. or carcinom$.mp. or leuk?emia.mp. or lymphom$.mp. or sarcom$.mp.  **5** 4 AND (1 OR 2 OR 3)  **6** limit 5 to english or limit 5 to german  **7** (6 and humans/) or (6 not animals/)  **8** (comprehensive* or integrative or systematic*) adj3 (bibliographic* or review* or literature)) or (meta-analy* or metaanaly* or "research synthesis" or ((information or data) adj3 synthesis) or (data adj2 extract*))).ti, ab. or (cinahl or (cochrane adj3 trial*) or embase or medline or psyclit or (psycinfo not "psycinfo database") or pubmed or scopus or "sociological abstracts" or "web of science" or central).ab. or ("cochrane database of systematic reviews" or evidence report technology assessment or evidence report technology assessment summary).jn. or Evidence Report: Technology Assessment*.jn. or (network adj1 analy*).ti, ab.) or (((review adj5 (rationale or evidence)).ti, ab. and review.pt.) or meta-analysis as topic/or Meta-Analysis.pt.)  **9** Randomi?ed controlled trial?.pt. or controlled clinical trial?.pt. or randomi?ed.ti, ab.or placebo.ti, ab. or drug therapy.sh. or randomly.ti, ab. or trial?.ti, ab. or group?.ti, ab.  **10** 7 AND (8 OR 9) |
| Ovid Embase | **1** exp cannabidiol OR exp cannabidivarin OR exp cannabinol OR exp cannabis OR exp dronabinol OR exp medical cannabis OR exp nabiximols OR exp tetrahydrocannabinol OR cannabis.mp OR cannabinoid$.mp OR cannabidiol.mp OR cannabinol.mp OR Mariuana.mp OR Marihuana.mp OR Marijuana.mp OR hemp.mp OR hanf.mp OR phytocannabinoid.mp. OR tetrahydrocannabinol.mp OR THC.mp OR CBD.mp OR tetrahydrocannabivarin.mp.  **2** nabiximols.mp OR sativex.mp OR dronabinol.mp OR marinol.mp OR namisol.mp OR nabilon.mp (sind dann in Kombi mit Krebs nur 3 Treffer, die nicht relevant sind)  **3** (synthetic adj1 cannabi$).mp OR (synthetic adj1 marijuana).mp OR (synthetic adj1 marihuana).mp OR K2.mp OR spice.mp  **4** exp neoplasm/or neoplasm$.mp or cancer$.mp. or tumo?r$.mp. or malignan$.mp. or oncolog$.mp. or carcinom$.mp. or leuk?emia.mp. or lymphom$.mp. or sarcom$.mp.  **5** (1 OR 2 OR 3) AND 4  **6** limit 5 to english or limit 5 to german  **7** (6 and humans/) or (6 not animals/)  **8** ((((comprehensive* or integrative or systematic*) adj3 (bibliographic* or review* or literature)) or (meta-analy* or metaanaly* or "research synthesis" or ((information or data) adj3 synthesis) or (data adj2 extract*))).ti, ab. or (cinahl or (cochrane adj3 trial*) or embase or medline or psyclit or (psycinfo not "psycinfo database") or pubmed or scopus or "sociological abstracts" or "web of science" or central).ab. or ("cochrane database of systematic reviews" or evidence report technology assessment or evidence report technology assessment summary).jn. or Evidence Report: Technology Assessment*.jn. or (network adj1 analy*).ti, ab.) or (exp Meta Analysis/or ((data extraction.ab. or selection criteria.ab.) and review.pt.))  **9** crossover procedure/or double blind procedure/or randomized controlled trial/or single blind procedure/or (random$ or factorial$ or crossover$ or (cross adj1 over$) or placebo$ or (doubl$ adj1 blind$) or (singl$ adj1 blind$) or assign$ or allocat$ or volunteer$).ti, ab, de.  **10** 7 AND (8 OR 9) |
| Cochrane | **#1** [mh Cannabinoids] OR [mh “Medical Marijuana”] OR cannabis OR cannabinoid* OR cannabidiol OR cannabinol OR Mariuana OR Marihuana OR Marijuana OR hemp OR hanf OR phytocannabinoid.mp. OR tetrahydrocannabinol OR THC OR CBD OR tetrahydrocannabivarin  **#2** nabiximols OR sativex OR dronabinol OR marinol OR namisol OR nabilon  **#3** (synthetic NEXT cannabi*) OR (synthetic NEXT marijuana) OR (synthetic NEXT marihuana) OR K2 OR spice  **#4** [mh neoplasms] or neoplasm* or cancer? or tum*r? or malignan* or oncolog* or carcinom* or leuk*mia or lymphoma? or sarcoma?  **#5 (#**1 OR #2 OR #3) AND #4 |
| Ebsco – PsychINFO | **S1** DE "Cannabis" OR DE "Cannabinoids" OR DE "Hashish" OR DE "Marijuana" OR DE  "Tetrahydrocannabinol" OR TX cannabis OR TX cannabinoid* OR TX cannabidiol OR TX cannabinol OR TX Mariuana OR TX Marihuana OR TX Marijuana OR TX hemp OR TX hanf OR TX phytocannabinoid OR TX tetrahydrocannabinol OR TX THC OR TX CBD OR TX tetrahydrocannabivarin OR DE “Medical Marijuana”  **S2** TX nabiximols OR TX sativex OR TX dronabinol OR TX marinol OR TX namisol OR TX nabilon  **S3** TX (synthetic N1 cannabi*) OR TX (synthetic N1 marijuana) OR TX (synthetic N1marihuana) OR TX K2 OR TX spice  **S4** ((DE "Neoplasms" OR DE "Benign Neoplasms" OR DE "Breast Neoplasms" OR DE "Endocrine Neoplasms" OR DE "Leukemias" OR DE "Melanoma" OR DE "Metastasis" OR DE "Nervous System Neoplasms" OR DE "Terminal Cancer") OR (TX neoplasm* OR TX cancer OR TX tumo#r OR TX malignan* OR DE "oncology“ OR TX oncolog* OR TX carcinom* OR TX leuk#emia OR TX lymphoma OR TX sarcoma))  **S5** S4 AND (S1 OR S2 OR S3)  **S6** (LA German OR LA English)  **S7** S5 AND S6  **S8** ((comprehensive* OR integrative OR systematic*) N3 (bibliographic* OR review* OR literature)) OR (meta-analy* or metaanaly* or "research synthesis" OR ((information OR data) N3 synthesis) OR (data N2 extract*)) OR ((review N5 (rationale OR evidence)) AND DE "Literature Review") OR (AB(cinahl OR (cochrane N3 trial*) OR embase OR medline OR psyclit OR pubmed OR scopus OR "sociological abstracts" OR "web of science" OR central)) OR DE "Meta Analysis" OR (network N1 analy*)  **S9** DE "Treatment Effectiveness Evaluation" OR DE "Treatment Outcomes" OR DE "Psychotherapeutic Outcomes" OR DE "Placebo" or DE "Followup Studies" OR placebo* OR random* OR "comparative stud*" OR (clinical N3 trial*) OR (research N3 design) OR (evaluat* N3 stud*) OR (prospectiv* N3 stud*) OR ((singl* OR doubl* OR trebl* OR tripl*) N3 (blind* OR mask*)  **S10** S7 AND (S8 OR S9) |
| Ebsco- CINAHL | **S1** MH Cannabis OR MH Medical Marijuana OR TX cannabis OR TX cannabinoid* OR TX cannabidiol OR TX cannabinol OR TX Mariuana OR TX Marihuana OR TX Marijuana OR TX hemp OR TX hanf OR TX phytocannabinoid OR TX tetrahydrocannabinol OR TX THC OR TX CBD OR TX tetrahydrocannabivarin  **S2** TX nabiximols OR TX sativex OR TX dronabinol OR TX marinol OR TX namisol OR TX nabilon (in Kombi mit Krebs, nurnoch 6 die nicht relevant sind)  **S3** TX (synthetic N1 cannabi*) OR TX (synthetic N1 marijuana) OR TX (synthetic N1marihuana) OR TX K2 OR TX spice  **S4** (MH "Neoplasms+" OR TX neoplasm* OR TX cancer OR TX tumo#r OR TX malignan* OR TX oncolog* OR TX carcinom* OR TX leuk#emia OR TX lymphoma OR TX sarcoma)  **S5** S4 AND (S1 OR S2 OR S3)  **S6** (LA German OR LA English)  **S7** S5 AND S6  **S8** (TI (systematic* n3 review*)) or (AB (systematic* n3 review*)) or (TI (systematic* n3 bibliographic*)) or (AB (systematic* n3 bibliographic*)) or (TI (systematic* n3 literature)) or (AB (systematic* n3 literature)) or (TI (comprehensive* n3 literature)) or (AB (comprehensive* n3 literature)) or (TI (comprehensive* n3 bibliographic*)) or (AB (comprehensive* n3 bibliographic*)) or (TI (integrative n3 review)) or (AB (integrative n3 review)) or (JN “Cochrane Database of Systematic Reviews”) or (TI (information n2 synthesis)) or (TI (data n2 synthesis)) or (AB (information n2 synthesis)) or (AB (data n2 synthesis)) or (TI (data n2 extract*)) or (AB (data n2 extract*)) or (TI (medline or pubmed or psyclit or cinahl or (psycinfo not “psycinfo database”) or “web of science” or scopus or embase)) or (AB (medline or pubmed or psyclit or cinahl or (psycinfo not “psycinfo database”) or “web of science” or scopus or embase or central)) or (MH “Systematic Review”) or (MH “Meta Analysis”) or (TI (meta-analy* or metaanaly*)) or (AB (meta-analy* or metaanaly*)) or network n1 analy*  **S9** (MH "Clinical Trials+") or PT Clinical trial or TX clinic* n1 trial* or TX ((singl* n1 blind*) or (singl* n1 mask*)) or TX ((doubl* n1 blind*) or (doubl* n1 mask*)) or TX ((tripl* n1 blind*) or (tripl* n1 mask*)) or TX ((trebl* n1 blind*) or (trebl* n1 mask*)) or TX randomi* control* trial* or (MH "Random Assignment") or TX random* allocat* or TX placebo* or MH "Placebos") or MH "Quantitative Studies") or TX allocat* random*  **S10** S7 AND (S8 OR S9) |

**Table S2: Excluded studies**

| **Author** | **Year** | **Titel** | **Reason for Exclusion** |
| --- | --- | --- | --- |
| Coté et al. | 2016 | Improving quality of life with nabilone during radiotherapy treatments for head and neck cancers: a randomized double-blind placebo-controlled trial. | Outcome does not match criteria |
| Strasser et al. | 2006 | Comparison of orally administered cannabis extract and delta-9-tetrahydrocannabinol in treating patients with cancer-related anorexia-cachexia syndrome: a multicenter, phase III, randomized, double-blind, placebo-controlled clinical trial from the Cannabis-In-Cachexia-Study-Group | Outcome does not match criteria |
| Hardy et al. | 2023 | Phase IIb randomized, placebo-controlled, dose-escalating, double-blind study of cannabidiol oil for the relief of symptoms in advanced cancer (MedCan1-CBD | Outcome does not match criteria |
| Johnson et al. | 2010 | Multicenter, double-blind, randomized, placebo-controlled, parallel-group study of the efficacy, safety, and tolerability of THC: CBD extract and THC extract in patients with intractable cancer-related pain. | Outcome does not match criteria |
| Lucraft et al. | 1982 | Randomised clinical trial of levonantradol and chlorpromazine in the prevention of radiotherapy-induced vomiting. | Outcome does not match criteria |
| Priestman et al. | 1987 | A double-blind randomised cross-over comparison of nabilone and metoclopramide in the control of radiation-induced nausea | Outcome does not match criteria |
| Niiranen & Mattson et al. [54] | 1987 | Antiemetic efficacy of nabilone and dexamethasone: a randomized study of patients with lung cancer receiving chemotherapy | Outcome does not match criteria |
| Lichtman et al. [55] | 2018 | Results of a Double-Blind, Randomized, Placebo-Controlled Study of Nabiximols Oromucosal Spray as an Adjunctive Therapy in Advanced Cancer Patients with Chronic Uncontrolled Pain | Outcome does not match criteria |
| Fallon et al. [56] | 2017 | Sativex oromucosal spray as adjunctive therapy in advanced cancer patients with chronic pain unalleviated by optimized opioid therapy: two double-blind, randomized, placebo-controlled phase 3 studies | Outcome does not match criteria |
| Portenoy et al. [57] | 2012 | Nabiximols for opioid-treated cancer patients with poorly controlled chronic pain: a randomized, placebo-controlled, graded-dose trial | Outcome does not match criteria |
| Jatoi et al. [58] | 2002 | Dronabinol *versus* megestrol acetate *versus* combination therapy for cancer-associated anorexia: a North Central Cancer Treatment Group study | Outcome does not match criteria |
| Zylla et al. [59] | 2021 | A randomized trial of medical cannabis in patients with stage IV cancers to assess feasibility, dose requirements, impact on pain and opioid use, safety, and overall patient satisfaction | Outcome does not match criteria |
| Twelves et al. [60] | 2021 | A phase 1b randomized, placebo-controlled trial of nabiximols cannabinoid oromucosal spray with temozolomide in patients with recurrent glioblastoma | Outcome does not match criteria |
| Turcott et al. [61] | 2018 | The effect of nabilone on appetite, nutritional status, and quality of life in lung cancer patients: a randomized, double-blind clinical trial | Outcome does not match criteria |
| Lynch et al. [62] | 2014 | A double-blind, placebo-controlled, crossover pilot trial with extension using an oral mucosal cannabinoid extract for treatment of chemotherapy-induced neuropathic pain | Outcome does not match criteria |
| Brisbois et al. [63] | 2011 | Delta-9-tetrahydrocannabinol may palliate altered chemosensory perception in cancer patients: results of a randomized, double-blind, placebo-controlled pilot trial | Outcome does not match criteria |
| Jatoi et al. [64] | 2002 | Does megestrol acetate downregulate interleukin-6 in patients with cancer-associated anorexia and weight loss? A North Central Cancer Treatment Group investigation | Outcome does not match criteria |
| Clarke et al. [65] | 2022 | Pilot clinical and pharmacokinetic study of DELTA9-Tetrahydrocannabinol (THC)/Cannabidiol (CBD) nanoparticle oro-buccal spray in patients with advanced cancer experiencing uncontrolled pain | Study type does not match criteria |
| Wanishpongpan & Oranratanaphan et al. [66] | 2022 | A randomized controlled study between THC cannabis oil and placebo added on standard prophylaxis for reducing chemotherapy-induced nausea vomiting (CINV) following carboplatin and paclitaxel regimen. | Publication type does not match criteria |
| Jahn et al. [67] | 2022 | The Prevention and Treatment of Nausea and Vomiting During Tumor Therapy | Publication type does not match criteria |

**Table S3: Risk of Bias Table**

| **Reference** | **Study type** | **Standardized rating of risk of bias** | **Additional comments on methodology** |
| --- | --- | --- | --- |
| Sukpiriyagul et al. (2023) | RCT | RoB  Domain 1: low  Domain 2: high concerns  Domain 3: some concerns  Domain 4: some concerns  Domain 5: some concerns  Overall risk of bias: high | PRO:  Sample: big sample size (n=60), adequate according to power analysis (n=54)  Methodological quality: ethics vote; baseline characteristics comparable; double blinding; power analysis was conducted; wash-out phase (> 21 days)  CONTRA:  Methodological quality: no ITT; analysis of nausea using an independent t test, although a test for dependent samples would be appropriate for a crossover design; no ANOVA results presented, although announced; the table only includes t tests; mention of testing for order effects, but no results presented graphically, the differences in the 2nd cycle are larger, suggesting the possibility of unblinding and/or potential bias due to order effects  Report quality: no details provided for dropout reasons |
| Grimison et al. (2020) | RCT | RoB  Domain 1: low  Domain 2: high  Domain 3: low  Domain 4: some concerns  Domain 5: low  Overall risk of bias: high | PRO:  Sample: big sample size (n=81), adequate according to power analysis  Methodological quality: ethics vote; power analysis was conducted; daily contact with staff on days of intake; structured collection of side effects using a checklist; adherence with diary and capsule counting; McNemar's test to control for within-patient correlation; control for order effects (p=0.29); comparability of groups ensured by study design  CONTRA:  Methodological quality: no ITT; no correction for multiple testing for secondary endpoints; no explanation why 90% CI (p=0.1) is used for primary endpoints and 95% CI is used for secondary endpoints; no distinction of the primary endpoint into acute or delayed, although this is listed in the methodology; unclear presentation of when and how each endpoint is measured; many contacts with the pharmaceutical industry; dosage of capsules individualized -> no subgroup analyses for high or low doses |
| Duran et al. (2010) | RCT | RoB  Domain 1: high  Domain 2: low  Domain 3: low  Domain 4: some concerns  Domain 5: low  Overall risk of bias: high | PRO:  Methodological quality: ethics vote; double blinding; ITT was conducted  CONTRA:  Sample: small sample size (n= 16), no power analysis  Methodological quality: no power analysis due to exploratory nature, but it was planned to recruit 60 participants; baseline difference in cancer type –> all in group (placebo) have breast cancer, group (THC:CBD) shows more variability; cancer extension also descriptively different –> all in group (olacebo) have localized cancer; baseline MANE and FLIE descriptively different –> likely not significant due to small group sizes; heterogeneous population (cancer type, chemotherapy)  Report quality: only a few p values were reported |
| Meiri et al. (2007) | RCT | RoB  Domain 1: high concerns  Domain 2: low  Domain 3: low  Domain 4: some concerns  Domain 5: low  Overall risk of bias: high | PRO:  Sample: big sample size (n= 64), not adequate according to power analysis  Methodological quality: ethics vote; double blinding; ITT was conducted; ANOVA; post hoc analysis; safety analysis; examination of compliance  CONTRA:  Methodological quality: no control for multiple testing for secondary endpoints; adherence to the medication regimen decreased during the study (per-protocol dose was adhered to by 45%); the study was supposed to recruit 464 patients –> underpowered; no exclusion of mixed effects, as all intervention groups received dronabinol on day 1  Report quality: few descriptive values for ECOG or QoL status; inadequate presentation of results, mostly through bar charts –> few statistical values (e.g. p values and SD), no transparency in the data provided |
| Lane et al. (1991) | RCT | RoB  Domain 1: Some concerns  Domain 2: high  Domain 3: low  Domain 4: some concerns  Domain 5: some concerns  Overall risk of bias: high | PRO:  Sample: big sample size (n=62), no power analysis  Methodological quality: No significant differences between the groups in baseline characteristics; compliance was monitored by counting pills every day  CONTRA:  Sample: small sample size and no power analysis  Methodological quality: no ethics approval; no ITT; not clear when the data were collected; Descriptive baseline age differences show that Group A had an average age of 47 years, Group B 49 years, and Group C 55.5 years, making Group C, on average, 8.5 years older than Group A; Group C had fewer patients with high emetogenicity of chemotherapy (16, 18, and 14). About half of the patients in each group had previously received prochlorperazine; there are no specific details about prior antiemetic therapy; most patients were also taking other medications, with varying numbers of agents (15, 17, and 12 for 2 or 3 agents); it is unclear how well the groups are comparable, as the distribution of other medications is not specified; particularly "younger" patients seem to have dropped out due to side effects; overall, there was a high dropout rate  Report quality: no specific details about the randomization; only graphical representations of the results are provided, where p values are noted, but they are poorly readable |
| McCabe et al. (1988) | RCT | RoB  Domain 1: Some concerns  Domain 2: low  Domain 3: low  Domain 4: high  Domain 5: some concerns  Overall risk of bias: high | CONTRA:  Sample: moderate sample size (n=36) and no power analysis  Methodological quality: no ethics vote; no ITT analysis or power analysis conducted; all patients experienced nausea and vomiting refractory to standard antiemetics; 34 had used prochlorperazine in the past; no double blinding  Report quality: all patients were evaluated and needed only at least one contact with either THC or prochlorperazine, but there is no description of how many rounds the patients completed |
| Pomeroy et al. (1986) | RCT | RoB  Domain 1: low  Domain 2: high  Domain 3: some concerns  Domain 4: some concerns  Domain 5: some concerns  Overall risk of bias: high | PRO:  Methodological quality: The number of patients receiving cisplatin-containing regimens and those receiving regimens without cisplatin is equally distributed between the groups  CONTRA:  Sample: moderate sample size (n=38) and no power analysis  Methodological quality: no ethics vote; no ITT analysis, one patient with continuous vomiting was excluded from the cycle 2 analysis; no comprehensive baseline comparison; 1n endpoint 1, a patient who continuously vomited in group (domperidone) was excluded.  Report quality: there were few details on blinding and randomization, and no information on whether blinding was effective, especially considering the more frequent side effects in group (nabilone); additionally, there were no details on whether patients received other antiemetics during the study, nor on how long a cycle lasts and the number of days between cycles |
| Niederle (1986) | RCT | RoB  Domain 1: low  Domain 2: some concerns  Domain 3: low  Domain 4: high  Domain 5: some concerns  Overall risk of bias: high | CONTRA:  Sample: small sample size (n=20) and no power analysis  Methodological quality: no ethics vote; no ITT analysis  Report quality: Graphs without precise numerical data |
| Crawford et al. (1986) | RCT | RoB  Domain 1: some concerns  Domain 2: high  Domain 3: low  Domain 4: some concerns  Domain 5: some concerns  Overall risk of bias: high | PRO:  Methodological quality: ethics vote  CONTRA:  Sample: moderate sample size (n=32) and no power analysis  Methodological quality: no ITT analysis; interventions administered at different times, making double-blinding questionable; few baseline characteristics provided -> age and gender of patients not given  Report quality: the age of the participants is not provided |
| Niiranen et al. (1985) | RCT | RoB  Domain 1: low  Domain 2: high  Domain 3: some concerns  Domain 4: some concerns  Domain 5: some concerns  Overall risk of bias: high | PRO:  Sample: big sample size (n=152) but no power analysis  Methodological quality: none of the statistical results indicate differences due to the order of interventions; patients did not take any other antiemetic medications during the study  CONTRA:  Sample: small sample size and no power analysis  Methodological quality: no ethics approval; no ITT analysis;  Report quality: discrepancy in dose specification: abstract: 2 mg and 15 mg, text: 1 mg and 7.5 mg, and no indication in the methods section if 2 tablets were given |
| Sheidler et al. (1984) | RCT | RoB  Domain 1: low  Domain 2: high  Domain 3: low  Domain 4: some concerns  Domain 5: some concerns  Overall risk of bias: high | PRO:  Sample: adequate according to power analysis  CONTRA:  Methodological quality: no ethics approval; there is no baseline statistics; the intervention duration per cycle was short (10 hours); the duration of medication administration does not adequately cover the duration of vomiting and nausea induced by chemotherapy; four patients (25%) required additional antiemetics in at least one cycle after the four doses of the study medication; there was no wash-out period, posing a risk of carryover effects or drug interactions; It is unclear if blinding was effective due to the numerous side effects.  Report quality: there is no information on whether patients took other antiemetics or had previously received Levonantradol or Prochlorperazine. Only categorical values for nausea/vomiting were reported, with no detailed individual data. There is no separate listing for individual phases |
| Heim et al. (1984) | RCT | RoB  Domain 1: some concerns  Domain 2: high  Domain 3: low  Domain 4: high  Domain 5: some concerns  Overall risk of bias: high | PRO:  Methodological quality: bonferroni correction  CONTRA:  Sample: big sample size (n=57) and no power analysis  Methodological quality: no ethics approval; no ITT analysis; no power analysis  Report quality: no information on whether there was a wash-out phase |
| Hutcheon et al. (1983) | RCT | RoB  Domain 1: low  Domain 2: low  Domain 3: low  Domain 4: some concerns  Domain 5: some concerns  Overall risk of bias: some concerns | PRO:  Sample: large sample size (n=108) but no power analysis  CONTRA:  Methodological quality: no ethics approval; of the 87 patients who showed side effects, 36 did not complete the study; there are no details on the duration of chemotherapy regimens, making it unclear on which days the data were collected and how often; group (levonantradol 0,75) contained a larger number of patients receiving cisplatin; it is unclear how dropouts, listed under adverse effects, were factored into the analysis, as all patients were evaluated  Report quality: there are no precise details on dropouts; all 108 patients are included in the analysis, but there is no information on how patients who did not complete the study were handled |
| Ahmedzai et al. (1983) | RCT | RoB  Domain 1: some concerns  Domain 2: high  Domain 3: low  Domain 4: some concerns  Domain 5: some concerns  Overall risk of bias: high | PRO:  Methodological quality: patients were not informed that Nabilone is cannabis-based  CONTRA:  Sample: moderate sample size (n=34) and no power analysis  Methodological quality: no ethics approval; no ITT analysis; no power analysis; In cases of severe nausea or vomiting despite study medication, parenteral administration of 10 mg metoclopramide or 50 mg chlorpromazine was given as needed; the volume of vomit was not measured according to a specific routine or at set intervals; all but four patients had not previously undergone chemotherapy; baseline difference at T0: a higher proportion in group (prochlorperazine) reported gagging |
| Kleinman et al. (1983) | RCT | RoB  Domain 1: low  Domain 2: high  Domain 3: low  Domain 4: some concerns  Domain 5: some concerns  Overall risk of bias: high | CONTRA:  Sample: small sample size (n=16) and no power analysis  Methodological quality: no ethics approval; no ITT analysis; no power analysis  Report quality: No detailed description of the study design |
| Jones et al. (1982) | RCT | RoB  Domain 1: low  Domain 2: high  Domain 3: high  Domain 4: some concerns  Domain 5: some concerns  Overall risk of bias: high | PRO:  Sample: big sample size (n=54) but no power analysis  Methodological quality: groups are comparable; safety analysis was conducted; no other antiemetics were allowed  CONTRA:  Methodological quality: no ethics approval; no ITT analysis; no power analysis  Report quality: unclear when data were collected, and which data were compared |
| Ungerleider et al. (1982) | RCT | Domain 1: some concerns  Domain 2: high  Domain 3: low  Domain 4: high  Domain 5: some concerns  Overall risk of bias: high | PRO:  Sample: big sample size (n=214) but no power analysis  Methodological quality: test for sequence effects  CONTRA:  Methodological quality: no ethics approval; no ITT analysis; no power analysis; patients who could guess when they received THC performed better with THC compared to those who could not guess (p < 0.05), sign.; of the 83% of patients who previously undergone chemotherapy, 29% reported that prochlorperazine was completely ineffective; approximately half of the patients had prior experience with marijuana |
| Johansson et al. (1982) | RCT | RoB  Domain 1: some concerns  Domain 2: high  Domain 3: some concerns  Domain 4: some concerns  Domain 5: some concerns  Overall risk of bias: high | PRO:  Methodological quality: patients with prior marijuana use were excluded  CONTRA:  Sample: small sample size (n=27) and no power analysis  Methodological quality: no ethics approval; no ITT or power analysis; when nabilone was given first, the difference was greater compared to prochlorperazine (endpoint 1); for endpoint 2, patients generally had worse scores in the second round; patients who received nabilone first had a mean of 6.7 episodes, while those who received nabilone second had a mean of 14.2 episodes; for patients who received prochlorperazine first, the mean was 18.8 episodes, and for those who received prochlorperazine second, it was 31.3 episodes  Report quality: no information on gender distribution; the text and table show different values for endpoint 4: 81% in the text and 72% in the table |
| Levitt et al. (1982) | RCT | RoB  Domain 1: some concerns  Domain 2: high  Domain 3: high  Domain 4: high  Domain 5: some concerns  Overall risk of bias: high | CONTRA:  Sample: big sample size (n=58) but no power analysis  Methodological quality: no ethics approval; no ITT analysis; no power analysis  Report quality: no precise information on the duration of each cycle or the number of days the chemotherapy is administered; no data on how many patients received each type of chemotherapy |
| Wada et al. (1982) | RCT | RoB  Domain 1: low  Domain 2: high  Domain 3: high  Domain 4: high  Domain 5: some concerns  Overall risk of bias: high | PRO:  Sample: big sample size (n=114) but no power analysis  CONTRA:  Methodological quality: no ethics approval; no ITT analysis; no power analysis; for endpoint 1 (vomiting): 5 patients were excluded because they vomited continuously (patients from group placebo)  Report quality: the group assignment of some dropouts is partially unclear |
| Neidhart et al. (1981) | RCT | RoB  Domain 1: low  Domain 2: high  Domain 3: some concerns  Domain 4: some concerns  Domain 5: some concerns  Overall risk of bias: high | CONTRA:  Sample: big sample size (n=77) but no power analysis  Methodological quality: no ethics approval; no ITT analysis; no power analysis; variability in the number of patients evaluated for different endpoints, as it was not required that all questions be answered  Report quality: No p values reported; no description of the statistical analysis; the crossover procedure is poorly described; no participant numbers are given for endpoints; only the summed rounds of subjects in a group are provided |
| Einhorn et al. (1981) | RCT | RoB  Domain 1: low  Domain 2: high  Domain 3: some concerns  Domain 4: some concerns  Domain 5: some concerns  Overall risk of bias: high | PRO:  Sample: big sample size (n=100) but no power analysis  Methodological quality: testing for carry-over effects  CONTRA:  Methodological quality: no ethics approval; no ITT analysis; no power analysis; study design was changed for the last 44 patients: they received 3 doses starting 12 hours before chemotherapy; different chemotherapy variants and varying numbers of days on which they were administered; no explanation provided to patients about what constitutes a "high"  Report quality: minimal baseline characteristics; with no breakdown by group unclear |
| Chang et al. (1981) | RCT | RoB  Domain 1: low  Domain 2: high  Domain 3: low  Domain 4: high  Domain 5: some concerns  Overall risk of bias: high | PRO:  Methodological quality: variation in inhaled THC was minimized through a standardized inhalation technique  CONTRA:  Sample: small sample size (n=9) and no power analysis  Methodological quality: no ethics approval; no ITT analysis; no power analysis  Report quality: No p values reported |
| Orr et al. (1981) | RCT | RoB  Domain 1: low  Domain 2: high  Domain 3: high  Domain 4: some concerns  Domain 5: some concerns  Overall risk of bias: high | PRO:  Sample: big sample size (n=24) but no power analysis  CONTRA:  Methodological quality: no ethics approval; no ITT analysis; no power analysis; 64% of patients who did not show a response received placebo as the first intervention, while 72% of patients who did show a response received THC first, and 36% received prochlorperazine first; few statistical comparisons or statistically relevant values provided -> overall difficult to interpret  Report quality: limited baseline information -> unclear if the groups are comparable; reasons for dropouts are poorly explained |
| Colls et al. (1980) | RCT | RoB  Domain 1: low  Domain 2: low  Domain 3: low  Domain 4: some concerns  Domain 5: some concerns  Overall risk of bias: some concerns | PRO:  Methodological quality: ethics approval  CONTRA:  Sample: moderate sample size (n=35) and no power analysis  Methodological quality: no ITT analysis; no power analysis  Report quality: patients are adults but no specific age details provided; indication: (solid tumors) not specified in detail |
| Sallan et al. (1980) | RCT | RoB  Domain 1: low  Domain 2: high  Domain 3: some concerns  Domain 4: high  Domain 5: some concerns  Overall risk of bias: high | PRO:  Sample: big sample size (n=84) and no power analysis  Methodological quality: analysis of age effects  CONTRA:  Methodological quality: no ethics approval; no ITT analysis; no power analysis; effectiveness and preference might depend on the type of chemotherapy the patient receives; only short-term effects assessed; no statistical comparisons of subgroups sorted by chemotherapy classes  Report quality: limited baseline information or clarity on group comparability; no data on the distribution of participants across the 6 groups; exclusion reasons cannot be attributed to specific groups. |
| Steel et al. (1980) | RCT | RoB  Domain 1: some concerns  Domain 2: high  Domain 3: high  Domain 4: high  Domain 5: some concerns  Overall risk of bias: high | PRO:  Sample: big sample size (n=55) and no power analysis  Methodological quality: Exclusion of patients who regularly used marijuana before the start of the study  CONTRA:  Methodological quality: no ethics approval; no ITT analysis; no power analysis; nabilone or prochlorperazine were given 15-18 hours before chemotherapy, leading patients to guess which medication they were receiving based on side effects; all 16 patients who believed they were receiving nabilone were correct (p < 0.05)  Report quality: limited baseline information (no details on age, gender, or type of cancer); no statistical values reported for significance |
| Herman et al. (1979) | RCT | RoB  Domain 1: low  Domain 2: high  Domain 3: low  Domain 4: high  Domain 5: some concerns  Overall risk of bias: high | PRO:  Sample: big sample size (n=152) but no power analysis  Methodological quality: Testing for sequence effects  CONTRA:  Methodological quality: no ethics approval; no ITT analysis; no power analysis; varying numbers of days for the intervention; different medication administration in Arizona and Indiana  Report quality: results are not reported separately by location; no reporting of means or other statistical values for nausea and vomiting; no baseline characteristics provided |
| Chang et al. (1979) | RCT | RoB  Domain 1: low  Domain 2: some concerns  Domain 3: low  Domain 4: some concerns  Domain 5: some concerns  Overall risk of bias: some concerns | PRO:  Methodological quality: variation in inhaled THC was minimized through a standardized inhalation technique; dose adjustments were made only for dysphoria, either orally or by smoking (reduced by 1/3); high compliance rate of 96%; testing for sequence effects.  CONTRA:  Sample: small sample size (n=15) and no power analysis  Methodological quality: no ethics approval; no ITT analysis; no power analysis;  majority of patients had prior experience with marijuan  Report quality: no baseline information provided; no comparison of group comparability |
| Kluin-Neleman et al. (1979) | RCT | RoB  Domain 1: low  Domain 2: high  Domain 3: low  Domain 4: some concerns  Domain 5: some concerns  Overall risk of bias: high | CONTRA:  Sample: small sample size (n=11) and no power analysis  Methodological quality: no ethics approval; no ITT analysis; no power analysis; 2 patients vomited after the 2nd and 3rd doses of THC  Report quality: last paragraph in the Discussion: the study had to be stopped because most patients experienced side effects and preferred to tolerate nausea and vomiting rather than continue the therapy |
| Frytak et al. (1975) | RCT | RoB  Domain 1: low  Domain 2: high  Domain 3: high  Domain 4: some concerns  Domain 5: some concerns  Overall risk of bias: high | PRO:  Sample: big sample size (n=117) but no power analysis  CONTRA:  Methodological quality: no ethics approval; no ITT analysis; no power analysis; patients who vomited more than twice were excluded from the study (n=18: THC n=10, prochlorperazine n=5, placebo n=3); some patients had to discontinue the study after the first day due to CNS toxicity or excessive vomiting  Report quality: discrepancy in dosage reporting: Abstract mentions THC 15 mg, while the main text reports 10 mg; no precise inclusion criteria provided |
| Sallan et al. (1975) | RCT | RoB  Domain 1: low  Domain 2: high  Domain 3: low  Domain 4: high  Domain 5: some concerns  Overall risk of bias: high | PRO:  Methodological quality: Effect of THC are independent of the order of interventions  CONTRA:  Sample: small sample size (n=22) and no power analysis  Methodological quality: no ethics approval; no ITT analysis; no power analysis  Report quality: no statistical comparisons for the entire sample; no baseline characteristics provided. |

ITT: Intention-to-treat; PP: per protocol; RCT: randomized controlled trial; small sample size: n<25; moderate sample size: n=25--50; large sample size: n=>5; Domain 1: randomized assignment; Domain 2: deviations from the intended interventions; Domain 3: missing outcome data; Domain 4: measurement of the outcome; Domain 5: selection of the reported result
